# Intratumoral presence of the genotoxic gut bacteria *pks*^+^ *E. coli*, Enterotoxigenic *Bacteroides fragilis*, and *Fusobacterium nucleatum* and their association with clinicopathological and molecular features of colorectal cancer

**DOI:** 10.1038/s41416-023-02554-x

**Published:** 2024-01-10

**Authors:** Jihoon E. Joo, Yen Lin Chu, Peter Georgeson, Romy Walker, Khalid Mahmood, Mark Clendenning, Aaron L. Meyers, Julia Como, Sharelle Joseland, Susan G. Preston, Natalie Diepenhorst, Julie Toner, Danielle J. Ingle, Norelle L. Sherry, Andrew Metz, Brigid M. Lynch, Roger L. Milne, Melissa C. Southey, John L. Hopper, Aung Ko Win, Finlay A. Macrae, Ingrid M. Winship, Christophe Rosty, Mark A. Jenkins, Daniel D. Buchanan

**Affiliations:** 1 Colorectal Oncogenomics Group, Department of Clinical Pathology, Victorian Comprehensive Cancer Centre, The University of Melbourne, Parkville, VIC Australia; 2 University of Melbourne Centre for Cancer Research, Victorian Comprehensive Cancer Centre, Parkville, VIC Australia; 3 Melbourne Bioinformatics, The University of Melbourne, Melbourne, VIC Australia; 4 Centre for Epidemiology and Biostatistics, Melbourne School of Population and Global Health, The University of Melbourne, Melbourne, VIC Australia; 5 Department of Microbiology and Immunology, The Peter Doherty Institute for Infection and Immunity, The University of Melbourne, Melbourne, VIC Australia; 6 Microbiological Diagnostic Unit Public Health Laboratory, Department of Microbiology and Immunology, The Peter Doherty Institute for Infection and Immunity, The University of Melbourne, Melbourne, VIC Australia; 7 Department of Infectious Diseases, Austin Health, Heidelberg, VIC Australia; 8 Endoscopy Unit, Department of Gastroenterology and Hepatology, The Royal Melbourne Hospital, Parkville, VIC Australia; 9 Melbourne Medical School, The University of Melbourne, Parkville, VIC Australia; 10 Cancer Epidemiology Division, Cancer Council Victoria, Melbourne, VIC Australia; 11 Precision Medicine, School of Clinical Sciences at Monash Health, Monash University, Melbourne, VIC Australia; 12 Department of Clinical Pathology, Melbourne Medical School, The University of Melbourne, Melbourne, VIC Australia; 13 Colorectal Medicine and Genetics, The Royal Melbourne Hospital, Parkville, VIC Australia; 14 Genomic Medicine and Family Cancer Clinic, Royal Melbourne Hospital, Parkville, Melbourne, VIC Australia; 15 Department of Medicine, The University of Melbourne, Parkville, VIC Australia; 16 Envoi Specialist Pathologists, Brisbane, QLD Australia; 17 University of Queensland, Brisbane, QLD Australia

**Keywords:** Oncogenesis, Risk factors, Cancer

## Abstract

**Background:**

This study aimed to investigate clinicopathological and molecular tumour features associated with intratumoral *pks*^*+*^ *Escherichia coli* (*pks*^*+*^*E.coli*^*+*^), *pks*^*+*^*E.coli*^*-*^ (non-*E.coli* bacteria harbouring the *pks* island), Enterotoxigenic *Bacteroides fragilis* (ETBF) and *Fusobacterium nucleatum* (*F. nucleatum*).

**Methods:**

We screened 1697 tumour-derived DNA samples from the Australasian Colorectal Cancer Family Registry, Melbourne Collaborative Cohort Study and the ANGELS study using targeted PCR.

**Results:**

*Pks*^*+*^*E.coli*^*+*^ was associated with male sex (*P* < 0.01) and *APC*:c.835-8 A > G somatic mutation (*P* = 0.03). The association between *pks*^*+*^*E.coli*^*+*^ and *APC*:c.835-8 A > G was specific to early-onset CRCs (diagnosed<45years, *P* = 0.02). The *APC*:c.835-A > G was not associated with *pks*^*+*^*E.coli*^*-*^ (*P* = 0.36). *F. nucleatum* was associated with DNA mismatch repair deficiency (MMRd), *BRAF:*c.1799T>A p.V600E mutation, CpG island methylator phenotype, proximal tumour location, and high levels of tumour infiltrating lymphocytes (*P*s < 0.01). In the stratified analysis by MMRd subgroups, *F. nucleatum* was associated with Lynch syndrome, *MLH1* methylated and double MMR somatic mutated MMRd subgroups (*Ps* < 0.01).

**Conclusion:**

Intratumoral *pks*^*+*^*E.coli*^*+*^ but not *pks*^*+*^*E.coli*^*-*^ are associated with CRCs harbouring the *APC*:c.835-8 A > G somatic mutation, suggesting that this mutation is specifically related to DNA damage from colibactin-producing *E.coli* exposures*. F. nucleatum* was associated with both hereditary and sporadic MMRd subtypes, suggesting the MMRd tumour microenvironment is important for *F. nucleatum* colonisation irrespective of its cause.

## Introduction

Colorectal cancer (CRC) was the third most prevalent cancer and the second leading cause of cancer-related death worldwide for both sexes in 2020 [1]. In Australia, it is the second most commonly diagnosed cancer with 1 in 21 (4.8%) and 1 in 30 (3.3%) males and females, respectively, developing CRC by 75 years of age [2]. CRC is a heterogeneous disease with regards to molecular drivers and pathways of tumorigenesis. In 2007, Jass et al. [3] described the molecular heterogeneity of CRC based on the presence or absence of chromosomal instability (CIN), microsatellite instability/mismatch repair deficiency (MSI/MMRd), CpG island methylator phenotype (CIMP), *BRAF* c.1799T>A p.Val600Glu (*BRAF* p.V600E), *KRAS* somatic mutations in codons 12 and 13 and germline pathogenic variants in the DNA MMR genes (Lynch syndrome). This heterogeneity over time has been shown to be associated with differing risk factors, survival, and treatment response. For example, serrated polyps, which are the precursors of the serrated pathway of tumorigenesis, characterised by *BRAF* p.V600E, CIMP-high and proximal location with or without high levels of MSI, show stronger association with tobacco smoking, alcohol intake and high body mass index (BMI) than conventional adenomas [4].

The etiology of the majority of CRC is multifactorial, involving the interplay between genetic, epigenetic, and environmental/lifestyle factors [5] whilst only ~5% of CRC are caused by germline pathogenic variants in known cancer-predisposition genes [6]. More recently, the gut microbiome is increasingly recognised to play an important role in CRC development [7, 8]. The presence of certain genotoxic gut bacteria is associated with CRC development [9]. Previous studies have shown that *pks*^*+*^ *Escherichia coli* (*pks*^*+*^ *E. coli*^*+*^), Enterotoxigenic *Bacteroides fragilis* (ETBF) and *Fusobacterium nucleatum* (*F. nucleatum*) are enriched in colonic mucosa of CRC-affected patients compared with healthy individuals [10–12].

*E. coli* strains from the B2 phylogroup [13] frequently harbour the 54 kb polyketide synthase (*pks*) island, which encodes enzymes for colibactin biosynthesis [14–16]. Colibactin is a genotoxin that induces DNA damage including inter-strand cross links [17] and double-stranded breaks [14, 18]. Recently, the colibactin-induced DNA damage was found to occur in specific patterns of single base substitution mutations (T > N), frequently in ATN and TTT sequence contexts [19]. This has led to the discovery of tumour mutational signatures associated with colibactin, in particular, the presence of single base substitution (SBS)-88 and short insertions and deletions (indels or ID)-18 signatures [19]. The somatic *APC* splice mutation (c.835-8 A > G) has been proposed as a biomarker of colibactin-induced DNA damage due to the specific sequence context of ATT > C and this has been observed in multiple adenomas from patients with unexplained colorectal polyposis though the presence of *pks* +* E.coli*^*+*^ was not measured [20].

ETBF secretes *Bacteroides fragilis* toxin and can cause symptoms such as inflammatory diarrhoea in humans [21]. ETBF triggers colitis, induces T_H_17 cell infiltration in murine models and promotes colonic tumorigenesis [22], although the mechanism underlying ETBF-related colorectal tumorigenesis is currently unknown.

*F. nucleatum* produces *F. nucleatum* adhesin A, which alters the β-catenin/Wnt signalling pathway and promotes CRC tumour growth [23]. *F. nucleatum* inhibits T-cell mediated immune responses against tumour cells [24] as well as creates a pro-inflammatory microenvironment that is favourable for colorectal neoplasia progression [25]. *F. nucleatum* is associated with CRCs that demonstrate MSI-high/MMRd, *BRAF* p.V600E somatic mutation and CIMP-high, features of the serrated pathways of tumorigenesis [12].

The aim of this study was to identify the intratumoral presence of genotoxic gut bacterial species, namely *pks*^*+*^ in *E. coli* (*pks*^*+*^ *E. coli*^*+*^), *pks*^*+*^ in non-*E. coli* (*pks*^*+*^ *E. coli*^−^) and ETBF, and *F. nucleatum*, in population-based CRC tumour samples from the Australasian Colorectal Cancer Family Registry (ACCFR) and Melbourne Collaborative Cohort Study (MCCS), and from the clinic-based CRC tumour samples from the Applying Novel Genomic approaches to Early-onset and suspected Lynch Syndrome colorectal and endometrial cancers (ANGELS) study. The association between the intratumoral bacteria and specific clinicopathological characteristics and molecular features, including the *APC* c.835-8 A > G somatic mutation were examined.

## Methods

### Australasian Colorectal Cancer Family Registry (ACCFR)

The ACCFR is the Australasian arm of the International Colon Cancer Family Registry with >42,000 recruited participants [26, 27]. Tumours tested in this study were from the population-based recruitment arm of the ACCFR, recruited from the Victorian Cancer Registry independent of family cancer history and diagnosed with invasive carcinoma of the colon or rectum between 1997 and 2007 during two recruitment phases [26]. Phase I recruitment (1997–2001) involved all CRC patients diagnosed between 18 and 44 years of age and 50% of CRC patients diagnosed between 45 and 59 years of age. Phase II recruitment (2001-2006) involved all CRC patients between 18 and 49 years of age [26]. From the Jeremy Jass Memorial Tissue Bank, a total of 823 primary adenocarcinomas of the colon or rectum during two recruitment phases with tumour tissue collected were available to this study [28]. Cancers were verified using obtainable pathology reports, cancer registry reports, medical records, and/or death certificates [26, 27].

### Melbourne Collaborative Cohort Study (MCCS)

The MCCS is a prospective cohort study composed of 41,513 participants - 17,044 males and 24,469 females- recruited between 1990 and 1994 [29, 30], designed to understand the role of diet and lifestyle associated with cancer risk, including CRC [29, 30]. Tumour tissue was collected and molecularly characterised for a total of 858 CRCs, with the diagnosis age ranging from 41 to 86 years [28].

### Applying Novel Genomic approaches to Early-onset and suspected Lynch Syndrome colorectal and endometrial cancers (ANGELS)

The ANGELS study recruited patients referred from family cancer clinics across Australia who were: (1) CRC- or endometrial cancer-affected people with an MMRd and/or microsatellite instability high (MSI-H) tumour with a diagnosis of suspected Lynch syndrome (as previously defined [31]), or (2) CRC- or endometrial cancer-affected people with an MMR-proficient (MMRp) and/or microsatellite stable cancer diagnosed <45 years of age. The ANGELS study had collected CRC tumour tissue for 229 participants diagnosed between 2014–2021 [32, 33].

Written informed consent was obtained from all study participants to collect blood and tumour tissue materials. The study protocols were approved by Human Research Ethics Committees at the University of Melbourne (ACCFR and ANGELS) and Cancer Council Victoria (MCCS). Given their rarity across the three study groups, CRCs from germline biallelic *MUTYH* pathogenic variant carriers (*n* = 4), constitutional *MLH1* epimutation carriers (*n* = 6) and germline carriers of variant of uncertain significance in the MMR genes (VUS, *n* = 9) were excluded from this study. The details for samples included in this study are shown in Supplementary Fig. 1. A total of 29 participants had synchronous and metachronous CRCs and individual CRCs were treated independently in the statistical analysis.

### Analyses of clinicopathological features and molecular characteristics

CRC tumour tissue was available for 813 and 816 probands for ACCFR and MCCS, respectively, as previously described [28], and 221 probands from the ANGELS study. A standardised pathological review was performed by anatomical pathologist (CR) for all three studies [34]. Tumours from the ileo-cecal junction, cecum, ascending colon, hepatic flexure, and transverse colon were classified as proximal, whereas tumours from the splenic flexure, descending, sigmoid colon and recto-sigmoid junction were grouped as distal. Tumours from the rectum were classified as rectal. Tumour-infiltrating lymphocytes were scored as present when there were ≥ 5 intra-epithelial lymphocytes in at least one high-power field (40×) [35].

Molecular characterisation of each tumour was performed using consistent methodology for the ACCFR and MCCS studies [28]. Tumour MMR status was determined by immunohistochemical (IHC) staining for *MLH1, MSH2, MSH6* and *PMS2* protein expression on all CRCs and for a subset, MSI status was determined as previously described [36–38]. For the ACCFR and MCCS studies, the primary antibodies used were MLH1 (G168-15, BD PharMingen), MSH2 (G219-1129, BD PharMingen), MSH6 (44, BD Transduction Labs) and PMS2 (A16-4, BD PharMingen). For the ANGELS study, tumour MMR status was determined from MMR IHC testing by clinical diagnostic laboratory or by MMR IHC testing performed internally as previously described [39] and confirmed by whole exome or targeted tumour sequencing using the additive feature combination approach [33]. For the ANGELS study, the primary antibodies used were MLH1 (M1), MSH2 (G219-1129), MSH6 (SP93) and PMS2 (A16-4) supplied from Roche Diagnostics (Basel, Switzerland). Across the three studies, tumours were categorised as: (1) MMRp if they showed retained and normal expression of all four MMR proteins by IHC and were microsatellite stable according to the additive feature combination approach or MSI-PCR method where tested, or (2) MMRd if they demonstrated loss of expression of one or more MMR proteins by IHC and/or were MSI-high according to the additive feature combination approach or MSI-PCR method where tested [28, 33].

MMRd CRCs were further divided into three subgroups: (1) Lynch syndrome - where a germline pathogenic variant in one of the DNA MMR genes (*MLH1*, *MSH2*, *MSH6* and *PMS2*) or in *EPCAM* was identified as previously described [26, 38, 40, 41]; (2) *MLH1* methylated CRCs - positive for tumour hypermethylation of the *MLH1* gene promoter using the MethyLight assay as previously described for ACCFR and MCCS [28, 40, 42] and using MethyLight and MS-HRM assays as previously described for ANGELS [39] with *MLH1* methylation positive tumours showing concomitant loss of *MLH1* and *PMS2* expression by IHC; (3) Somatic biallelic MMR gene inactivation resulting from two somatic MMR gene mutations (double somatic MMR mutations) determined as described previously from either targeted tumour sequencing assay [39, 43] or from tumour whole exome sequencing [32, 33].

For the ACCFR and MCCS, *KRAS* codons 12&13 somatic mutations were tested using real-time quantitative PCR (qPCR) with high-resolution melting analysis in the presence of the SYTO9 fluorescent intercalating dye followed by direct Sanger sequencing on cases with differential melting profiles as previously described [34, 44]. *BRAF p*.V600E somatic mutation was tested using a fluorescent allele-specific polymerase chain reaction assay as previously described [45]. For the ANGELS study, *KRAS* codons 12&13 and *BRAF* p.V600E somatic mutations were derived from custom-designed panel sequencing or tested using Sanger or allele-specific PCR as for the ACCFR/MCCS tumours [32, 33, 39]. CIMP-high tumours were defined by tumour hypermethylation at 3 or more of the promoter regions of the 5 tumour suppressor genes: *CACNA1G, IGF2, NEUROG1, RUNX3* and *SOCS1* using MethyLight [46] for all three studies.

### DNA extraction from tumour samples and qPCR assays for detecting *pks*^*+*^*E. coli, ETBF and F. nucleatum*

The tumour rich regions of the FFPE CRC tumour tissue were macrodissected as previously described for the ACCFR and MCCS studies [28, 43]. For the ANGELS study, the genomic DNA was extracted from FFPE CRC tumour tissues using the QIAamp DNA FFPE Tissue Kit (QIAGEN, Hilden, Germany), and the concentration was assessed using the Qubit fluorometer (Thermo Fisher Scientific, California, USA). The intratumoral presence of *pks*^*+*^ *E. coli*^*+*^, *pks*^*+*^ *E. coli*^−^, ETBF, and *F. nucleatum* was assessed by performing qPCR, which is detailed in Supplementary methods. The *pks*^*+*^ *E. coli* strains (34351) were kindly provided by Drs Danielle Ingle and Norelle Sherry from the Peter Doherty Institute for Infection and Immunity, collected as part of the Controlling Superbugs study [47] and classed as Extraintestinal Pathogenic *Escherichia coli* (ExPEC) [48]. The *pks*^*-*^ *E. coli* strains (MG1655) were provided by Dr. Dianna M. Hocking (Department of Microbiology and Immunology, The University of Melbourne). Genomic DNA from *pks*^*+*^ *E. coli* and *pks- E. coli* was used as internal controls for qPCR.

### Genotyping assay for the *APC* c.835-8 A > G somatic mutation

A custom-designed TaqMan genotyping assay was used to detect the *APC*: c.835-8 A > G mutation in tumour DNA (ThermoFisher Scientific, California, USA, Cat# ANGZYCC), which was set up using TaqMan Genotyping Master Mix (ThermoFisher Scientific), and performed on Thermo QuantStudio 7 (ThermoFisher Scientific). The presence of *APC*: c.835-8 A > G mutation was determined using the QuantStudio Real-time PCR System software (v1.7.2, ThermoFisher Scientific).

### Statistical analysis

All statistical tests were performed using R programming software (v4.2.1). Logistic regression was used to assess the association between the presence of intratumoral bacteria and clinicopathological and tumour features. Unless indicated otherwise, all tests were adjusted for sex, age at CRC diagnosis and study. *P* < 0.05 were considered statistically significant.

## Results

### Participant and tumour characteristics associated with the intratumoral bacterial presence

In total, 1697 CRC tumours from 1666 individuals from the ACCFR (44.2%), MCCS (43.5%), and ANGELS (12.3%) had results from intratumoral bacteria testing. A description of the participants and their tumour characteristics are provided in Table 1. Of those, 29 (1.7%) participants had a synchronous or metachronous CRC, 26% were diagnosed with CRC before age 45 years (early-onset CRC or EOCRC) and 15.6% were MMRd. Of these MMRd CRCs with an explained etiology, 20.6% were related to Lynch syndrome while the remaining 79.4% were related to somatic MMR inactivation with 47.4% related to tumour *MLH1* methylation and 32% related to double somatic MMR mutations.

**Table 1:** CRC-affected participants and their tumour characteristics from each of the three studies.

Characteristics	Total	ACCFR	MCCS	ANGELS
Total CRC patients	1666	736 (44.2%)	725 (43.5%)	205 (12.3%)
Numbers of CRC patients with synchronous and metachronous CRCs	29	5	20	4
Sex				
Female	824 (49.5%)	356 (48.4%)	351 (48.4%)	117 (57.1%)
Male	842 (50.5%)	380 (51.6%)	374 (51.6%)	88 (42.9%)
Total CRCs^a^	1697	742 (43.7%)	745 (43.9%)	210 (12.4%)
Age at CRC diagnosis				
Mean ± IQR	55.7 ± 24.8	46.5 ± 11	68.5 ± 11.4	44.1 ± 19.5
Early-onset CRC (<45 years)	442 (26%)	301 (40.6%)	4 (0.5%)	137 (65.2%)
Late-onset CRC (≥45 years)	1255 (74%)	441 (59.4%)	741 (99.5%)	73 (34.8%)
MMR status				
MMR-proficient (MMRp)	1432 (84.4%)	669 (90.2%)	645 (86.6%)	118 (56.2%)
MMR-deficient (MMRd)	265 (15.6%)	73 (9.8%)	100 (13.4%)	92 (43.8%)
Anatomical location				
Proximal	576 (33.9%)	201 (27.1%)	267 (35.8%)	108 (51.4%)
Distal	467 (27.5%)	214 (28.8%)	196 (26.3%)	57 (27.2%)
Rectal	582 (34.3%)	288 (38.8%)	249 (33.4%)	45 (21.4%)
Unknown	72 (4.3%)	39 (5.3%)	33 (4.5%)	0 (0%)
*BRAF* somatic mutation				
* BRAF* V600E	197 (11.6%)	53 (7.1%)	124 (16.7%)	24 (11.4%)
Wildtype	1420 (83.7%)	682 (91.9%)	603 (80.9%)	149 (71%)
Unknown	80 (4.7%)	7 (1%)	18 (2.4%)	37 (17.6%)
*KRAS* somatic mutation				
* KRAS* codon 12&13 somatic mutation	461 (27.2%)	211 (28.4%)	203 (27.3%)	51 (24.3%)
Wildtype	1088 (64.1%)	451 (60.8%)	529 (71%)	122 (58.1%)
Unknown	148 (8.7%)	80 (10.8%)	13 (1.7%)	37 (17.6%)
CIMP				
CIMP-high	171 (10.1%)	35 (4.7%)	109 (14.6%)	27 (12.9%)
CIMP-negative	1399 (82.4%)	613 (82.6%)	624 (83.8%)	162 (77.1%)
Unknown	127 (7.5%)	94 (12.7%)	12 (1.6%)	21 (10%)
Tumour histological type				
Adenocarcinoma	1437 (84.7%)	654 (88.2%)	653 (87.6%)	130 (61.9%)
Mucinous	143 (8.4%)	64 (8.6%)	59 (7.9%)	20 (9.5%)
Other type	22 (1.3%)	6 (0.8%)	13 (1.8%)	3 (1.5%)
Unknown	95 (5.6%)	18 (2.4%)	20 (2.7%)	57 (27.1%)
Tumour grade				
High	345 (20.3%)	141 (19%)	143 (19.2%)	61 (29%)
Low	1301 (76.7%)	577 (77.8%)	581 (78%)	143 (68.1%)
Unknown	51 (3%)	24 (3.2%)	21 (2.8%)	6 (2.9%)
Tumour-infilteration lymphocytes (TILs)				
Present	373 (22%)	147 (19.8%)	170 (22.8%)	56 (26.7%)
Absent	1195 (70.4%)	558 (75.2%)	538 (72.2%)	99 (47.1%)
Unknown	129 (7.6%)	37 (5%)	37 (5%)	55 (26.2%)
MMRd CRC Subgroup^b^				
Lynch syndrome	47 (20.6%)	37 (58.8%)	6 (7.4%)	4 (4.8%)
* MLH1* methylation	108 (47.4%)	13 (20.6%)	71 (87.7%)	24 (28.5%)
Double somatic MMR mutations^c^	73 (32%)	13 (20.6%)	4 (4.9%)	56 (66.7%)

^a^Includes synchronous and metachronous CRCs from same individuals.

^b^MMRd CRCs with unexplained causes are not shown.

^c^Double MMR somatic pathogenic mutations identified using NGS-based techniques.

The prevalence of each of the bacteria and their association with participant clinicopathological and tumour characteristics are shown in Table 2. Intratumoral prevalence of *pks*^*+*^ *E. coli*^*+*^*, pks*^*+*^ *E. coli*^*−*^, ETBF and *F. nucleatum* was 10.3%, 10.4%, 6.1% and 8.9%, respectively. CRCs with intratumoral *pks*^*+*^ *E. coli*^*+*^, were associated with male sex (*P* < 0.01, odds ratio (OR) = 1.54, 95% confidence interval (CI) = 1.11–2.13) when compared with female sex. CRCs with intratumoral *pks*^*+*^ *E. coli*^*−*^ were associated with older age at CRC diagnosis *(P* < 0.01) and low-grade tumours when compared with high-grade tumours (*P* = 0.02, OR^high grade^ = 0.57, 95% CI = 0.36–0.89).

**Table 2:** Intratumoural presence of *pks+ E. coli*, *pks+ E. coli*^*−*^, *ETBF and F nucleatum* and the association with tumour characteristics.

	*pks*^*+*^ *E.coli*^*+*^					*pks*^*+*^ *E.coli*^*-*^					Enterotoxigenic *Bacteroides fragilis*					*Fusobacterium nucleatum*				
Characteristics	Detected	Not detected	Unknown	*P* value^a^	Adjusted Odd Ratio^a^ (95% CI)	Detected	Not detected	Unknown	*P* value^a^	Adjusted Odd Ratio^a^ (95% CI)	Detected	Not detected	Unknown	*P* value^a^	Adjusted Odd Ratio^a^ (95% CI)	Detected	Not detected	Unknown	*P* value^a^	Adjusted Odd Ratio^a^ (95% CI)
Total CRCs	174 (10.3%)	1339 (78.9%)	184 (10.8%)	-	-	176 (10.4%)	1337 (78.8%)	184 (10.8%)	-	-	104 (6.1%)	1451 (85.5%)	142 (8.4%)	-	-	151 (8.9%)	1440 (84.9%)	106 (6.2%)	-	-
Recruitment^b^				0.4306	-				0.2157	-				0.5240	-				**0.0024**	**-**
Population-based	152 (10.2%)	1170 (78.7%)	165 (11.1%)	-	Ref	162 (10.9%)	1160 (78%)	165 (11.1%)	-	Ref	93 (6.3%)	1258 (84.6%)	136 (9.1%)	-	Ref	**120 (8.1%)**	**1266 (85.1%)**	**101 (6.8%)**	**-**	**Ref**
Clinic-based	22 (10.5%)	169 (80.5%)	19 (9%)	-	1.22 (0.73–1.98)	14 (6.7%)	177 (84.3%)	19 (9%)	-	0.69 (0.37–1.20)	11 (5.2%)	193 (91.9%)	6 (2.9%)	-	0.80 (0.39–1.51)	**31 (15%)**	**174 (83%)**	**5 (2%)**	**-**	**2.03 (1.27–3.17)**
Sex				**0.009701**	-				0.6114	-				0.6181	-				0.2619	-
Female	**67 (8%)**	**660 (79%)**	**109 (13%)**	**-**	Ref	86 (10.3%)	641 (76.7%)	109 (13%)	-	Ref	49 (6%)	723 (86%)	64 (8%)	-	Ref	69 (8.3%)	720 (86.1%)	47 (5.6%)	-	Ref
Male	**107 (12.4%)**	**679 (78.9%)**	**75 (8.7%)**	**-**	**1.54 (1.11–2.13)**	90 (10.5%)	696 (80.8%)	75 (8.7%)	-	0.92 (0.67–1.27)	55 (6.4%)	728 (84.5%)	78 (9.1%)	-	1.11 (0.74–1.66)	82 (9.5%)	720 (83.6%)	59 (6.9%)	-	1.21 (0.87–1.71)
Age (mean ± IQR)	57.8 ± 26.0	55.3 ± 24.3	58.0 ± 22.1	0.27052	-	**58.8** **±** **22.7**	**55.2** **±** **25.1**	**58.0** **±** **22.1**	**0.0061**	**-**	56.7 ± 25.9	55.8 ± 25.1	55.3 ± 20.2	0.8806	-	55.8 ± 27.0	56.0 ± 25.1	54.4 ± 21.4	0.7854	-
Age group^c^				0.8462	-				0.0533	-				0.2520					0.4110	-
LOCRC (≥45)	132 (10.5%)	977 (77.9%)	146 (11.6%)	-	Ref	144 (11.5%)	965 (76.9%)	146 (11.6%)	-	Ref	75 (6%)	1071 (85.3%)	109 (8.7%)	-	Ref	105 (8.4%)	1071 (85.3%)	79 (6.3%)	-	Ref
EOCRC (<45)	42 (9.5%)	362 (81.9%)	38 (8.6%)	-	1.05 (0.67–1.63)	32 (7.2%)	372 (84.2%)	38 (8.6%)	-	0.63 (0.40–1.00)	29 (7%)	380 (86%)	33 (7%)	-	1.38 (0.79–2.42)	46 (10.4%)	369 (83.5%)	27 (6.1%)	-	1.21 (0.77–1.91)
CRC site																				
Proximal colon	62 (10.8%)	457 (79.3%)	57 (9.9%)	-	Ref	61 (10.6%)	458 (79.5%)	57 (9.9%)	-	Ref	44 (7.6%)	485 (84.2%)	47 (8.2%)	-	Ref	**85 (14.8%)**	**457 (79.3%)**	**34 (5.9%)**	**-**	Ref
Distal colon	49 (10.5%)	371 (79.4%)	47 (10.1%)	0.9924	1.00 (0.66–1.50)	49 (10.5%)	371 (79.4%)	47 (10.1%)	0.9232	1.02 (1.53–0.68)	24 (5.1%)	404 (86.5%)	39 (8.4%)	0.0862	0.63 (0.37–1.06)	**31 (7%)**	**406 (87%)**	**30 (6%)**	**8.22E-05**	**0.42 (0.26–0.64)**
Rectum	55 (9.5%)	460 (79%)	67 (11.5%)	0.5165	0.88 (0.59–1.30)	54 (9.3%)	461 (79.2%)	67 (11.5%)	0.5203	0.88 (1.31–0.59)	31 (5%)	506 (87%)	45 (8%)	0.0708	0.64 (0.39–1.03)	**34 (6%)**	**514 (88%)**	**34 (6%)**	**3.00E-06**	**0.36 (0.23–0.55)**
Unknown	8 (11.1%)	51 (70.8%)	13 (18.1%)	-	-	12 (16.7%)	47 (65.3%)	13 (18%)	-	-	5 (6.9%)	56 (77.8%)	11 (15.3%)	-	-	**1 (1.4%)**	**63 (87.5%)**	**8 (11.1%)**	**-**	**-**
MMR status				0.5656	-				0.1588	-				**0.0021**	**-**				**7.33E-12**	**-**
MMRp	148 (10.3%)	1122 (78.4%)	162 (11.3%)	-	Ref	155 (10.8%)	1115 (77.9%)	162 (11.3%)	-	Ref	**78 (5.5%)**	**1232 (86%)**	**122 (8.5%)**	**-**	Ref	**94 (6.6%)**	**1249 (87.2%)**	**89 (6.2%)**	**-**	Ref
MMRd	26 (9.8%)	217 (81.9%)	22 (8.3%)	-	0.87 (0.53–1.37)	21 (7.9%)	222 (83.8%)	22 (8.3%)	-	0.69 (0.41–1.13)	**26 (9.8%)**	**219 (82.6%)**	**20 (7.6%)**	**-**	**2.16 (1.30–3.49)**	**57 (21.5%)**	**191 (72.1%)**	**17 (6.4%)**	**-**	**3.90 (2.63–5.75)**
*BRAF* somatic mutation				0.4789	-				0.1297	-				0.5399	-				**0.0008**	**-**
Wildtype	149 (10.4%)	1127 (78.6%)	158 (11%)	-	Ref	154 (10.7%)	1122 (78.3%)	158 (11%)	-	Ref	91 (6.4%)	1215 (84.7%)	128 (8.9%)	-	Ref	**113 (7.9%)**	**1225 (85.4%)**	**96 (6.7%)**	**-**	Ref
V600E	18 (9%)	162 (80.6%)	21 (10.4%)	-	0.83 (0.48–1.36)	16 (8%)	164 (81.6%)	21 (10.4%)	-	0.65 (0.36–1.10)	11 (5.5%)	179 (89%)	11 (5.5%)	-	0.82 (0.40–1.50)	**31 (15.4%)**	**162 (80.6%)**	**8 (4%)**	**-**	**2.13 (1.35–3.28)**
Unknown	7 (11.3%)	50 (80.6%)	5 (8.1%)	-	-	6 (9.7%)	51 (82.2%)	5 (8.1%)	-	-	2 (3.2%)	57 (91.9%)	3 (4.9%)	-	-	**7 (11.3%)**	**53 (85.5%)**	**2 (3.2%)**	**-**	**-**
*KRAS* somatic mutation				0.5568	-				0.2526	-				**0.0178**	**-**				0.2901	-
Wildtype	110 (10%)	874 (79.3%)	118 (10.7%)	-	Ref	113 (10.3%)	871 (79%)	118 (10.7%)	-	Ref	**59 (5.4%)**	**958 (86.9%)**	**85 (7.7%)**	**-**	Ref	102 (9.3%)	933 (84.7%)	67 (6%)	-	Ref
codon 12&13	50 (10.8%)	360 (77.4%)	55 (11.8%)	-	1.11 (0.77–1.58)	56 (12%)	354 (76.2%)	55 (11.8%)	-	1.22 (0.86–1.72)	**39 (8.4%)**	**384 (82.6%)**	**42 (9%)**	**-**	**1.67 (1.09–2.53)**	35 (7.6%)	401 (86.2%)	29 (6.2%)	-	0.80 (0.53–1.19)
Unknown	14 (10.8%)	105 (80.8%)	11 (8.5%)	-	-	7 (5.4%)	112 (86.1%)	11 (8.5%)	-	-	**6 (4.6%)**	**109 (83.9%)**	**15 (11.5%)**	**-**	**-**	14 (10.8%)	106 (81.5%)	10 (7.7%)	-	-
CIMP status				0.8502	-				0.9232	-				0.6695	-				**3.71E-05**	**-**
CIMP-negative	149 (11%)	1098 (78%)	152 (11%)	-	Ref	144 (10.3%)	1103 (78.8%)	152 (10.9%)	-	Ref	90 (6.4%)	1187 (84.9%)	122 (8.7%)	-	Ref	**112 (8%)**	**1198 (85.6%)**	**89 (6.4%)**	**-**	Ref
CIMP-high	18 (10.5%)	135 (79%)	18 (10.5%)	-	0.95 (0.54–1.58)	19 (11.1%)	134 (78.4%)	18 (10.5%)	-	0.97 (0.56–1.61)	10 (5.8%)	152 (88.9%)	9 (5.3%)	-	0.86 (0.41–1.64)	**31 (18.1%)**	**131 (76.6%)**	**9 (5.3%)**	**-**	**2.62 (1.64–4.10)**
Unknown	7 (5.5%)	106 (83.5%)	14 (11%)	-	-	13 (10%)	100 (79%)	14 (11%)	-	-	4 (3.1%)	112 (88.2%)	11 (8.7%)	-	-	**8 (6.3%)**	**111 (87.4%)**	**8 (6.3%)**	**-**	**-**
CRC tumour grade				0.2109	-				**0.0161**	**-**				0.2760	-				**4.79E-05**	**-**
Low grade	140 (11%)	1012 (78%)	149 (11%)	-	Ref	**148 (11%)**	**1004 (77%)**	**149 (12%)**	**-**	**Ref**	76 (5.8%)	1119 (86%)	106 (8.2%)	-	Ref	**97 (7.5%)**	**1127 (86.6%)**	**77 (5.9%)**	**-**	Ref
High grade	30 (8.7%)	288 (83.5%)	27 (7.8%)	-	0.77 (0.50–1.15)	**24 (7%)**	**294 (85.2%)**	**27 (7.8%)**	**-**	**0.57 (0.36–0.89)**	25 (7.2%)	289 (83.8%)	31 (9%)	-	1.30 (0.80–2.06)	**51 (15%)**	**270 (78%)**	**24 (7%)**	**-**	**2.14 (1.48–3.08)**
Unknown	4 (7.8%)	39 (76.5%)	8 (15.7%)	-	-	**4 (7.8%)**	**39 (76.5%)**	**8 (15.7%)**	**-**	**-**	3 (5.9%)	43 (84.3%)	5 (9.8%)	-	-	**3 (5.9%)**	**43 (84.3%)**	**5 (9.8%)**	**-**	**-**
Tumour histological type																				
Adenocarcinoma	145 (10.1%)	1133 (78.8%)	159 (11.1%)	-	Ref	149 (10%)	1129 (79%)	159 (11%)	-	Ref	85 (5.9%)	1225 (85.3%)	127 (8.8%)	-	Ref	**111 (7.7%)**	**1229 (85.5%)**	**97 (6.8%)**	**-**	Ref
Mucinous	18 (12.6%)	111 (77.6%)	14 (9.8%)	0.4023	1.26 (0.72–2.09)	18 (12.6%)	111 (77.6%)	14 (9.8%)	0.4660	1.22 (0.70–2.03)	14 (9.8%)	120 (83.9%)	9 (6.3%)	0.0851	1.69 (0.89–2.98)	**26 (18.2%)**	**112 (78.3%)**	**5 (3.5%)**	**0.0001**	**2.51 (1.54–3.97)**
Other types	3 (13.6%)	19 (86.4%)	0 (0%)	0.7714	1.20 (0.28–3.61)	3 (13.6%)	19 (86.4%)	0 (0%)	0.8090	1.16 (0.27–3.50)	2 (9.1%)	19 (86.4%)	1 (4.5%)	0.5889	1.50 (0.24–5.31)	**4 (18.2%)**	**18 (81.8%)**	**0 (0%)**	**0.1265**	**2.37 (0.67–6.53)**
Unknown	8 (8.4%)	76 (80%)	11 (11.6%)	-	-	6 (6.3%)	78 (82.1%)	11 (11.6%)	-	-	3 (3%)	87 (92%)	5 (5%)	-	-	**10 (10.5%)**	**81 (85.3%)**	**4 (4.2%)**	**-**	**-**

^a^*P*-values and odd ratios were calculated using multiple logistic regression adjusted for CRC diagnosis age, sex and study cohort. Samples with unknown intratumoural presences and tumour characteristics treated as na and excluded from the statistical testing.

^b^Adjusted for sex and age only.

^c^Adjusted for sex and study cohort only.

Statistically significant *p* < 0.05 values are in bold.

CRCs with intratumoral ETBF were more likely to be MMRd (*P* < 0.01, OR = 2.16, 95% CI = 1.30–3.49) and have *KRAS* codons 12&13 somatic mutations (*P* = 0.02, OR = 1.67, 95% CI = 1.09–2.53) when compared with MMRp and CRCs without *KRAS* codon 12&13 somatic mutations, respectively. Intratumoral *F. nucleatum* was associated with proximal tumour location when compared with distal (*P* < 0.01, OR^distal^ = 0.42, 95% CI = 0.26–0.64) and rectal locations (*P* < 0.01, OR^rectal^ = 0.36, 95% CI = 0.23–0.55). CRCs with intratumoral *F. nucleatum* were also associated with high histology grade (*P* < 0.01, OR = 2.14, 95% CI = 1.48–3.08) and mucinous adenocarcinoma histologic type (*P* < 0.01, OR = 2.51, 95% CI = 1.54–3.97). For molecular features, intratumoral *F. nucleatum* was associated with CRCs with MMRd (*P* < 0.01, OR = 3.90, 95% CI = 2.63–5.75), *BRAF* p.V600E somatic mutation (*P* < 0.01, OR = 2.13, 95% CI = 1.35–3.28), and CIMP-high (*P* < 0.01, OR = 2.62, 95%CI = 1.64–4.10) when compared with CRCs without these features.

An analysis of the 442 EOCRCs did not show evidence that clinicopathological or tumour characteristics were associated with the presence of *pks*^*+*^ *E. coli*^*+*^ (Supplementary Table 1). In EOCRCs, the presence of ETBF (*P* < 0.01, OR = 4.17, 95% CI = 1.77–9.67) or *F. nucleatum* (*P* < 0.01, OR = 3.36, 95%CI = 1.67–6.65) was both associated with MMRd when compared with MMRp CRCs. *F. nucleatum* was also associated with the proximal tumour location when compared with distal tumour location (*P* = 0.01, OR^distal^ = 0.36, 95% CI = 0.16–7.70) and rectal tumour location (*P* < 0.01, OR^rectal^ = 0.33, 95% CI = 0.15–7.00) (Supplementary Table 1). ETBF was associated with proximal tumour location when compared with distal location (*P* = 0.04, OR^prox^ = 2.78/OR^distal^ = 0.36, 95%CI^prox^ = 0.13–6.25/95%CI^dist^ = 0.16–7.70) but this was not significant when compared with rectal location (*P* = 0.20, OR^prox^ = 3.03/OR^rectal^ = 0.33, 95%CI^prox^ = 0.14–6.67/95%CI^rectal^ = 0.15–7.00).

The presence of any two or all three of the bacteria (*pks*^*+*^ *E. coli*^*+*^, ETBF and *F. nucleatum*) in the same CRC was uncommon, with only 54 (3.2%) tumours having the presence of >1 bacteria (Supplementary Fig. 2). Only 6 (0.4%) tumours were detected with all three bacteria and these CRCs were not associated with any specific clinicopathological characteristics (Supplementary Table 2). CRCs that had both ETBF and *F. nucleatum* detected were associated with MMRd when compared with CRCs that did not have both ETBF and *F. nucleatum* detected (*P* < 0.01, OR = 4.56, 95%CI = 1.38–13.56) (Supplementary Table 2).

### The colibactin-associated APC: c.835-8 A > G somatic mutation is associated with pks^+^ E. coli^+^

The association between the intratumoral presence of the *pks* island, with or without *E. coli* (*pks*^*+*^ *E. coli*^*+*^ and *pks*^*+*^ *E. coli*^*−*^) and the *APC*: c.835-8 A > G somatic mutation was tested. Due to lower DNA requirement than the intratumoral bacterial screening, 62 additional samples (total n = 1759) were included in the *APC*: c.835-8 A > G testing. Across all CRCs, 3.3% had the *APC:* c.835-8 A > G somatic mutation, which was consistent with the frequency observed in both EOCRCs and late-onset CRCs (LOCRCs) (Table 3). The *APC:* c.835-8 A > G mutation was associated with intratumoral *pks*^*+*^ *E. coli*^*+*^ (*P* = 0.025, OR = 2.20, 95% CI = 1.05–4.25) but not with other bacteria carrying the *pks* island (*pks*^*+*^ *E. coli*^*−*^*; P* = 0.36, OR = 0.61, 95% CI = 0.18–1.54) or with the *E. coli* bacteria not carrying the *pks* island (*pks*^*-*^ *E. coli*^*+*^*; P* = 0.16, OR = 1.99, 95% CI = 0.67–4.72) (Table 3). These trends were consistent when tested in the EOCRCs (*P* = 0.022, OR = 4.26, 95%CI = 1.10–14.02), however, in the LOCRCs, the association between *APC*: c.835-8 A > G and *pks*^*+*^ *E. coli*^*+*^ was not significant (*P* = 0.18, OR = 1.78, 95% CI = 0.70–3.94) (Table 3). The *APC:* c.835-8 A > G mutation was not associated with intratumoral ETBF or *F. nucleatum* (data not shown). The participant and tumour characteristics associated with the *APC:* c.835-8 A > G somatic mutation are shown in Supplementary Table 3.

**Table 3:** The prevalence of the *APC:* c.835-8 A > G somatic mutation and its association with the intratumoral presence of *pks+ E. coli+*, pks+ E. coli^−^, all pks+ bacteria and pks- E. coli^+^.

	All CRCs				EOCRCs (CRC < 45 yrs)				LOCRCs (CRC ≥ 45 yrs)			
Intratumoural bacteria	*APC*: c.835-8 A > G mutation	*APC*: c.835-8 A > G wildtype	*P* value^a^	Adjusted Odd Ratio (95% CI)^a^	*APC*: c.835-8 A > G mutation	*APC*: c.835-8 A > G wildtype	*P* value^a^	Adjusted Odd Ratio (95% CI)^a^	*APC*: c.835-8 A > G mutation	*APC*: c.835-8 A > G wildtype	*P* value^a^	Adjusted Odd Ratio (95% CI)^a^
Total CRCs	58 (3.3%)	1701 (96.7%)	-	-	15 (3.3%)	439 (96.7%)	-	-	43 (3.3%)	1262 (96.7%)	-	-
*pks* + *E. coli* +			**0.0252**	**-**			**0.0219**	**-**			0.1820	**-**
Not detected	**41 (3.1%)**	**1287 (96.9%)**	**-**	**Ref**	**9 (2.5%)**	**351 (97.5%)**	**-**	**Ref**	32 (3.3%)	936 (96.7%)	-	Ref
Detected	**11 (6.3%)**	**163 (93.7%)**	**-**	**2.20 (1.05–4.25)**	**4 (9.5%)**	**38 (90.5%)**	**-**	**4.26 (1.10–14.02)**	7 (5.3%)	125 (94.7%)	-	1.78 (0.70–3.94)
Unknown	**6 (2.3%)**	**251 (97.7%)**	**-**	**-**	**2 (3.8%)**	**50 (96.2%)**	**-**	**-**	4 (2%)	201 (98%)	-	-
*pks* + *E. coli*^*−*^			0.3570	-			0.9931	-			0.6098	-
Not detected	48 (3.6%)	1279 (96.4%)	-	Ref	13 (3.5%)	357 (96.5%)	-	-	35 (3.7%)	922 (96.3%)	-	-
Detected	4 (2.3%)	171 (97.7%)	-	0.61 (0.18–1.54)	0 (0%)	32 (100%)	-	nt^b^	4 (2.8%)	139 (97.2%)	-	0.76 (0.22–1.95)
Unknown	6 (2.3%)	251 (97.7%)	-	-	2 (3.8%)	50 (96.2%)	-	-	4 (2%)	201 (98%)	-	-
*pks* + (all bacteria)			0.3775	-			0.2537	-			0.6323	-
Not detected	37 (3.1%)	1150 (96.9%)	-	Ref	9 (2.7%)	327 (97.3%)	-	Ref	28 (3.3%)	823 (96.7%)	-	Ref
Detected	15 (4%)	360 (96%)	-	1.32 (0.69–2.40)	4 (5.2%)	73 (94.8%)	-	2.03 (0.53–6.54)	11 (3.7%)	287 (96.3%)	-	1.19 (0.56–2.37)
Unknown	6 (3%)	191 (97%)	-	-	2 (4.9%)	39 (95.1%)	-	-	4 (2.6%)	152 (97.4%)	-	-
*pks- E. coli* +			0.1574	-			0.8141	-			0.0900	-
Not detected	47 (3.3%)	1374 (96.7%)	-	Ref	12 (3.2%)	365 (96.8%)	-	Ref	35 (3.4%)	1009 (96.6%)	-	Ref
Detected	5 (6.2%)	76 (93.8%)	-	1.99 (0.67–4.72)	1 (4%)	24 (96%)	-	1.29 (0.07–7.07)	4 (7.1%)	52 (92.9%)	-	2.56 (0.74–6.82)
Unknown	6 (2.3%)	251 (97.7%)	-	-	2 (3.8%)	50 (96.2%)	-	-	4 (2%)	201 (98%)	-	-

^a^Multiple logistic regression adjusted for CRC diagnosis age, sex and study cohort. Samples with unknown intratumoural presences and tumour characteristics were excluded in the statistical testing.

^b^Not tested as deemed non-sensical due to the group containing zero variable.

Statistically significant *p* < 0.05 values are in bold.

### F. nucleatum is associated with both inherited and sporadic subtypes of MMRd CRC

ETBF and *F. nucleatum* were associated with MMRd CRCs (Table 2). The etiology of MMRd for 228 of these CRCs was known comprising 47 (20.6%) CRCs from people with Lynch syndrome, and 181 (79.4%) related to sporadic causes namely *MLH1* promoter methylation (*n* = 108; 47.4%) and double somatic MMR mutations (*n* = 73; 32%). We further investigated the association between these bacteria and specific MMRd subgroups. The presence of *F. nucleatum* was associated with all three MMRd subgroups (Table 4), where the association was strongest for the *MLH1* methylated subgroup (*P* < 0.01, OR = 4.91, 95% CI = 2.84–8.36). No associations were observed between the specific MMRd subgroups and intratumoral *pks* + *E. coli* or ETBF, though ETBF was overall associated with MMRd status (Table 4).

**Table 4:** Intratumoural presence of pks+ E. coli, pks+ E. coli^−^, *ETBF and F nucleatum and* the association with CRC subgroup.

	*pks*^*+*^*E.coli*^*+*^					*pks*^*+*^*E.coli*^*-*^					Entrotoxigenic Bacteroides fragilis					Fusobacterium nucleatum				
CRC Subgroups	Detected	Not detected	Unknown	*P* value^a^	Adjusted Odd Ratio (95% CI)	Detected	Not detected	Unknown	*P* value^a^	Adjusted Odd Ratio (95% CI)	Detected	Not detected	Unknown	*P value*^a^	Adjusted Odd Ratio (95% CI)	Detected	Not detected	Unknown	*P* value^a^	Adjusted Odd Ratio (95% CI)
Total CRCs	171 (10.3%)	1311 (79.1%)	176 (10.6%)	*-*	*-*	173 (10.4%)	1309 (79%)	176 (10.6%)	-	-	95 (5.7%)	1425 (86%)	138 (8.3%)	*-*	*-*	146 (8.8%)	1411 (85.1%)	101 (6.1%)	*-*	*-*
Sporadic MMRp CRC	148 (10.4%)	1120 (78.3%)	162 (11.3%)	Ref	Ref	155 (11%)	1113 (78%)	162 (11%)	Ref	Ref	78 (5.5%)	1230 (86%)	122 (8.5%)	Ref	Ref	94 (6.6%)	1247 (87.2%)	89 (6.2%)	Ref	Ref
MMRd CRC subgroups^b^																				
Total MMRd CRCs with known etiology	23	191	14	***-***	***-***	18	196	14	-	-	-	195	16	*-*	*-*	52	164	12	*-*	*-*
Lynch syndrome	5 (11%)	41 (87%)	1 (2%)	0.8786	1.08 (0.36–2.58)	2 (4.3%)	44 (93.6%)	1 (2.1%)	0.1993	0.39 (0.06–1.30)	3 (6.4%)	39 (83%)	5 (10.6%)	0.6113	1.37 (0.32–3.96)	9 (19.2%)	33 (70.2%)	5 (10.6%)	**0.0016**	3.54 (1.53–7.46)
*MLH1* methylated	10 (9%)	87 (81%)	11 (10%)	0.4442	0.76 (0.35–1.48)	8 (7.4%)	89 (82.4%)	11 (10.2%)	0.1102	0.53 (0.23–1.09)	8 (7.4%)	92 (85.2%)	8 (7.4%)	0.5325	1.29 (0.54–2.71)	26 (24%)	76 (70%)	6 (6%)	**6.84** **×** **10**^**-9**^	4.91 (2.84–8.36)
Double somatic MMR mutations^c^	8 (11%)	63 (86.3%)	2 (2.7%)	0.7074	0.85 (0.34–1.93)	8 (11%)	63 (86.3%)	2 (2.7%)	0.4687	1.39 (0.54–3.28)	6 (8.2%)	64 (87.7%)	3 (4.1%)	0.2100	1.93 (0.64–5.12)	17 (23.3%)	55 (75.3%)	1 (1.4%)	**0.0002**	3.75 (1.85–7.39)

^a^Multiple logistic regression adjusted for CRC diagnosis age, sex and study cohort. Samples with unknown intratumoural presences and tumour characteristics were excluded in the statistical testing^.^

^b^Excludes MMRd CRCs with unexplained causes, primary and de novo *MLH1* epimutation carriers.

^c^Double MMR somatic pathogenic mutations identified using NGS-based techniques.

Statistically significant *p* < 0.05 values are in bold.

### Tumour-infiltrating lymphocytes (TILs) are associated with F. nucleatum but not with pks^+^ E. coli or ETBF

The intratumoral presence of each of *pks*^*+*^ *E. coli*^*+*^, *pks*^*+*^ *E. coli*^−^, ETBF, and *F. nucleatum* bacteria and the association with mild or marked levels of TILs present (combined as TILs present) within the tumour microenvironment was tested. The presence of TILs was associated with *F. nucleatum* (*P* < 0.01, OR = 1.97, 95% CI = 1.35–2.85), but not with *pks*^*+*^ *E. coli* or ETBF when tested across all CRCs (Table 5). The MMRd status was associated with both *F. nucleatum* (*P* < 0.01; Table 2) and TILs (*P* < 0.01; Table 5), we performed a stratified analysis to test whether the association between TILs and *F. nucleatum* is independent of MMR status. When the CRCs were stratified by tumour MMR status, the association between TILs and *F. nucleatum* was no longer present for either MMRp or MMRd CRCs (Table 5). These findings were consistent in both EOCRC and LOCRC (Table 5). The *APC*: c.835-8 A > G somatic mutation showed an inverse association with the presence of TILs across all CRCs (*P* < 0.01, OR^*APC*:c.835-8A>G^ = 0.19, 95% CI = 0.05–0.53), however, this observation was no longer significant when only MMRp CRCs were included in the analysis (*P* = 0.056, OR^*APC*:c.835-8A>G^ = 0.32, 95% CI = 0.08–0.87) (Supplementary Table 4).

**Table 5:** The association between tumour infiltrating lymphocytes (TILs) and intratumoral presences *pks+ E. coli, ETBF and F nucleatum*.

Intratumoral bacteria	All CRCs					EOCRCs (CRC < 45 yrs)					LOCRCs (CRC ≥ 45 yrs)				
	TILs present^a^	TILs absent	TILs unknown	*P* value^b^	Adjusted Odd Ratio (95% CI)	TILs present^a^	TILs absent	TILs unknown	*P* value^b^	Adjusted Odd Ratio (95% CI)	TILs present^a^	TILs absent	TILs unknown	*P* value^b^	Adjusted Odd Ratio (95% CI)
All CRCs															
pks+ E. coli negative	300 (22.4%)	934 (69.8%)	105 (7.8%)	-	Ref	81 (22.4%)	235 (64.9%)	46 (12.7%)	-	Ref	219 (22.4%)	699 (71.5%)	59 (6%)	-	Ref
pks+ E. coli+ detected	34 (19.5%)	131 (75.3%)	9 (5.2%)	0.2216	0.78 (0.51-1.15)	11 (26.2%)	28 (66.7%)	3 (7.1%)	0.8785	1.06 (0.48–2.2)	23 (17.4%)	103 (78%)	6 (4.5%)	0.1002	0.66 (0.40–1.06)
pks+ E. coli unknown	39 (21.2%)	130 (70.6%)	15 (8.2%)	-	-	9 (24%)	24 (63%)	5 (13%)	-	-	30 (20.5%)	106 (72.6%)	10 (6.8%)	-	-
ETBF negative	315 (21.7%)	1023 (70.6%)	113 (7.7%)	-	Ref	81 (21.3%)	248 (65.3%)	51 (13.4%)	-	Ref	234 (21.8%)	775 (72.4%)	62 (5.8%)	-	Ref
ETBF positive	28 (27%)	67 (64.4%)	9 (8.6%)	0.1887	1.36 (0.85-2.14)	11 (37.9%)	18 (62.1%)	0 (0%)	0.1743	1.75 (0.76–3.87)	17 (22.7%)	49 (65.3%)	9 (12%)	0.6984	1.12 (0.61–1.97)
ETBF unknown	30 (21.1%)	105 (74%)	7 (4.9%)	-	-	9 (27.3%)	21 (63.6%)	3 (9.1%)	-	-	21 (19.3%)	84 (77.1%)	4 (3.7%)	-	-
Fn negative	**300 (20.8%)**	**1032 (71.7%)**	**107 (7.5%)**	**-**	Ref	**76 (20.6%)**	**247 (66.9%)**	**46 (12.5%)**	**-**	**Ref**	**224 (20.9%)**	**785 (73.3%)**	**62 (5.8%)**	**-**	**Ref**
Fn positive	**51 (34%)**	**86 (57.3%)**	**13 (8.7%)**	**0.0004**	**1.97 (1.35–2.85)**	**18 (39.1%)**	**23 (50%)**	**5 (10.9%)**	**0.0154**	**2.33 (1.16–4.62)**	**33 (31.4%)**	**63 (60%)**	**9 (8.6%)**	**0.0235**	**1.70 (1.06–2.67)**
Fn unknown	**22 (20.8%)**	**77 (72.6%)**	**7 (6.6%)**	**-**	**-**	**7 (25.9%)**	**17 (63%)**	**3 (11.1%)**	**-**	**-**	**15 (19%)**	**60 (75.9%)**	**4 (5.1%)**	**-**	**-**
MMR status															
MMRp CRCs, *n* (%)	**215 (15.0%)**	**1129 (78.8%)**	**88 (6.2%)**	-	-	56 (15.4%)	271 (74.5%)	37 (10.1%)	-	-	159 (14.9%)	858 (80.3%)	51 (4.8%)	-	-
MMRd CRCs, *n* (%)	**158 (59.8%)**	**66 (25%)**	**40 (15.2%)**	2 × 10^−16^	12.57 (9.15–17.44)	45 (57.7%)	16 (20.5%)	17 (21.8%)	1.2 × 10^−15^	13.61 (7.33–26.46)	113 (60.4%)	50 (26.7%)	24 (12.8%)	2 × 10^−16^	12.20 (8.44–17.84)
MMRp CRCs only															
pks+ E. coli negative	171 (15%)	881 (79%)	69 (6%)	-	Ref	44 (14.8%)	223 (74.8%)	31 (10.4%)	-	Ref	127 (15.4%)	658 (79.9%)	39 (4.7%)	-	Ref
pks+ E. coli positive	19 (12.8%)	123 (83.1%)	6 (4.1%)	0.3079	0.77(0.45–1.25)	5 (16.1%)	24 (77.4%)	2 (6.5%)	0.9504	0.97 (0.31–2.52)	14 (12%)	99 (84.6%)	4 (3.4%)	0.1999	0.68 (0.36–1.19)
pks+ E. coli unknown	25 (15.4%)	125 (77.2%)	12 (7.4%)	-	-	7 (20%)	24 (68.6%)	4 (11.4%)	-	-	18 (14.2%)	101 (79.5%)	8 (6.3%)	-	-
ETBF negative	188 (15.3%)	968 (78.6%)	75 (6.1%)	-	Ref	49 (15.3%)	236 (73.5%)	36 (11.2%)	-	Ref	139 (15.3%)	732 (80.4%)	40 (4.4%)	-	Ref
ETBF positive	10 (12.8%)	61 (78.2%)	7 (9%)	0.5959	0.83 (0.39–1.58)	3 (17.6%)	14 (82.4%)	0 (0%)	0.9142	1.07 (0.24–3.50)	7 (11.5%)	47 (77%)	7 (11.5%)	0.4645	0.74 (0.30–1.57)
ETBF unknown	17 (13.9%)	100 (82%)	5 (4.1%)	-	-	4 (15.4%)	21 (80.8%)	1 (3.8%)	-	-	13 (13.5%)	79 (82.3%)	4 (4.2%)	-	-
Fn negative	189 (15.1%)	982 (78.7%)	77 (6.2%)	-	Ref	48 (15.2%)	234 (74.3%)	33 (10.5%)	-	Ref	141 (15.1%)	748 (80.1%)	45 (4.8%)	-	Ref
Fn positive	15 (16%)	74 (78.7%)	5 (5.3%)	0.8544	1.06 (0.57–1.83)	5 (17.9%)	20 (71.4%)	3 (10.7%)	0.6890	1.24 (0.39–3.30)	10 (15.2%)	54 (81.8%)	2 (3%)	0.9470	0.98 (0.46–1.89)
Fn unknown	11 (12.4%)	73 (82%)	5 (5.6%)	-	-	3 (14%)	17 (81%)	1 (5%)	-	-	8 (11.8%)	56 (82.4%)	4 (5.9%)	-	-
MMRd CRCs only															
pks+ E. coli negative	129 (60%)	53 (25%)	34 (15%)	-	Ref	37 (57.8%)	12 (18.8%)	15 (23.4%)	-	Ref	92 (60.1%)	41 (26.8%)	20 (13.1%)	-	Ref
pks+ E. coli positive	15 (57.7%)	8 (30.8%)	3 (11.5%)	0.6230	0.79 (0.32–2.10)	6 (54.5%)	4 (36.4%)	1 (9.1%)	0.3000	0.47 (0.11–2.11)	9 (60%)	4 (26.7%)	2 (13.3%)	0.7740	1.21 (0.34–4.97)
pks+ E. coli unknown	14 (64%)	5 (22%)	3 (14%)	-	-	2 (66.7%)	0 (0%)	1 (33.3%)	-	-	12 (63.2%)	5 (26.3%)	2 (10.5%)	-	-
ETBF negative	127 (58.3%)	55 (25.2%)	36 (16.5%)	-	Ref	32 (54.2%)	12 (20.4%)	15 (25.4%)	-	Ref	95 (59.4%)	43 (26.9%)	22 (13.8%)	-	Ref
ETBF positive	18 (69.2%)	6 (23.1%)	2 (7.7%)	0.7060	1.21 (0.46–3.58)	8 (66.7%)	4 (33.3%)	0 (0%)	0.7410	0.79 (0.19–3.57)	10 (71.4%)	2 (14.3%)	2 (14.3%)	0.4180	1.94 (0.46–13.36)
ETBF unknown	13 (65%)	5 (25%)	2 (10%)	-	-	5 (71.4%)	0 (0%)	2 (28.6%)	-	-	8 (61.5%)	5 (38.5%)	0 (0%)	-	-
Fn negative	111 (58.1%)	50 (26.2%)	30 (15.7%)	-	Ref	28 (52%)	13 (24%)	13 (24%)	-	Ref	83 (60.6%)	37 (27%)	17 (12.4%)	-	Ref
Fn positive	36 (64.3%)	12 (21.4%)	8 (14.3%)	0.4440	1.33 (0.65–2.88)	13 (72.2%)	3 (16.7%)	2 (11.1%)	0.3060	2.12 (0.55–10.55)	23 (59%)	9 (23.1%)	7 (17.9%)	0.7120	1.18 (0.50–2.95)
Fn unknown	11 (64.7%)	4 (23.5%)	2 (11.8%)	-	-	4 (66.7%)	0 (0%)	2 (33.3%)	-	-	7 (63.6%)	4 (36.4%)	0 (0%)	-	-

^a^Marked and mild tumour infiltrating lymphocytes were grouped to TILs present.

^b^Multiple logistic regression adjusted for CRC diagnosis age, sex and study cohort. Samples with unknown intratumoural presences and tumour characteristics were excluded in the statistical testing.

Statistically significant *p* < 0.05 values are in bold.

## Discussion

In this study of three Australian-based CRC cohorts comprising 1697 CRCs, the intratumoral prevalence of the *pks*^*+*^ *E. coli*^*+*^, ETBF and *F. nucleatum* genotoxic gut bacteria was 10%, 6% and 9%, respectively. The prevalence of other non-*E. coli* bacteria harbouring the pks island (*pks*^*+*^ *E. coli*^*−*^) was 10%. An association between the *APC*: c.835-8 A > G somatic mutation and the intratumoral presence of *pks*^*+*^ *E. coli*^*+*^ was observed, although no association was observed between this somatic mutation and other bacteria harbouring the *pks* island that produces the genotoxin colibactin, highlighting the specific relationship between this hotspot mutation and *pks*^*+*^ *E. coli*^*+*^ bacteria. The presence of ETBF or *F. nucleatum* were each associated with MMRd as was the presence of both of these bacteria in the same CRC. The co-occurrence of all three bacteria in the same CRC was uncommon (0.4% of all CRCs), suggesting there is minimal interplay between these bacteria at the time of CRC diagnosis.

### Pks^+^ E. coli

Colibactin-producing *pks*^*+*^ *E. coli*^*+*^ promotes CRC development by causing double-stranded DNA breakage [49, 50] and a specific pattern of mutational signature, namely SBS88 and ID18 [19]. The association between *APC*: c.835-8 A > G hotspot mutation and SBS88 have been identified in people with unexplained adenomatous polyposis [20], providing a mechanistic link and a potential biomarker for colibactin-induced tumorigenesis. This present study identified a significant association between the *APC*: c.835-8 A > G mutation and intratumoral *pks*^*+*^ *E. coli*^*+*^ but not with other *pks* harbouring bacteria (*pks*^*+*^ *E. coli*^*−*^), indicating the specific association of this mutation with *pks*^*+*^ *E. coli*^*+*^.

This study found that the *pks*^*+*^ *E. coli*^*+*^ tumours with the *APC*: c.835-8 A > G mutation were more prevalent in EOCRCs than LOCRCs (9.5% versus 5.3%), with only the association in EOCRCs showing statistical significance. The reason for this association in the EOCRCs is currently unknown and, if validated, raises interesting questions regarding the mechanism in EOCRC versus LOCRC. It has been hypothesised that early-life exposure to *pks*^*+*^ *E. coli*^+^ may influence early-onset tumorigenesis. *Pks*^*+*^ *E. coli*^*+*^ is a common gut bacteria found in ~31% of healthy infants by 1-month post-birth [51]. In addition, Lee-six et al. found that the colibactin-related mutation signature in normal colonic crypts from healthy young individuals and this was most active in younger children before reaching 10 years of age [52]. Therefore, the association between *APC*: c.835-8 A > G and *pks*^*+*^ *E. coli*^*+*^ in EOCRCs could be related to early-life exposure to the bacteria when our gut microbiome is still undergoing developmental changes [53], posing an especially “sensitive” period to extrinsic influences. Boot et al. argued that in later life when microbiome homoeostasis is established, individuals may be less susceptible to colibactin-related mutagenesis [54]. Studies aimed at prevention of colibactin-related EOCRC may need to focus on detection and eradication of *pks*^*+*^ *E. coli*^+^ in children.

In this study, *APC*: c.835-8 A > G mutation was detected in 3.3% of CRCs, a similar frequency (3.2%) to the previous report [55]. Whilst *APC*: c.835-8 A > G had a significant association with *pks*^*+*^ *E. coli*^*+*^, only a small subset (6.3%) of CRCs with *pks*^*+*^ *E. coli*^*+*^ had this mutation. As mentioned above, there might be a specific window when colibactin-induced damage is likely to occur, hence not causing the mutation in all CRCs exposed to the bacteria. Alternatively, prolonged exposure to this bacteria may be necessary to result in DNA damage. Studies suggest that up to 31% of healthy infants harbour *pks*^*+*^ *E. coli*^*+*^ by 1-month post-birth [51], though there still is no longitudinal study to investigate how long *pks*^*+*^ *E. coli*^*+*^ persists into the adult life and potentially induces CRC. Our study has focused on intratumoral *pks*^*+*^ *E. coli*^*+*^ at CRC diagnosis/resection and does not exclude prior *pks*^*+*^ *E. coli*^*+*^ infection. It is plausible that the association between *APC*: c.835-8 A > G and *pks*^*+*^ *E. coli*^*+*^ may be dependent on the duration of the exposure. Terlouw et al. [20], identified the *APC*: c.835-8 A > G mutation in premalignant adenomas from people with unexplained adenomatous polyposis supporting this mutation and colibactin-induced DNA damage as an early event in tumorigenesis, although further studies are needed to help elucidate this bacteria’s driver role during CRC development.

A recent study by Arima et al. [56] investigated intratumoral *pks*^*+*^ *E. coli* in 1175 CRCs collected as a part of two large prospective cohort studies. The authors found that intratumoral *pks*^*+*^ *E. coli* is associated with high western diet score, highlighting the interplay between gut bacterial pathogens and diet in CRC development. Consistent with the results reported by Arima et al. [56], this current study found no association between intratumoral *pks*^*+*^ *E. coli*^*+*^, and age at CRC diagnosis, tumour location, *BRAF* p.V600E mutation and CIMP-high. However, our study identified a significant association of *pks*^*+*^ *E. coli*^*+*^ with male sex, where this was not detected in Arima et al. Our study measured *pks*^*+*^ *E. coli*^*+*^ using two target genes for the *pks* island (C*lbB*) and *E. coli* (*UidA*), only capturing the presence of *pks*^*+*^ *E. coli*^+^, whereas Arima et al. [56] targeted only the *pks* island (*ClbB*) and, therefore, could not differentiate between *pks*^+^ in *E. coli* or *pks*^+^ in other bacteria. This may explain the association with the male gender, which was not present in our *pks* only analysis. In our study, there was no evidence of an association between *pks*^*+*^ *E. coli*^*+*^ and presence of elevated TILs, indicating that *pks*^*+*^ *E. coli*^*+*^ does not cause a highly immunogenic tumour microenvironment (TME), at least at the time of CRC diagnosis/resection.

### ETBF

ETBF was significantly enriched in MMRd CRCs when compared with MMRp CRCs but not associated with any specific MMRd subgroups of either hereditary or sporadic etiology. This suggests that ETBF may be associated with the tumour microenvironment related to MMRd, rather than playing a causative role in CRC development. ETBF is infectious bacteria, which cause acute inflammation of the colon and shown to be a risk factor for colitis [57]. ETBF is present in colonic mucosa of people with familial adenomatous polyposis [58] and promotes oncogenic processes in a tumour-prone mice model (*Apc*^*MinΔ/+*^) [59].

Allen et al. reported that ETBF promotes loss of heterozygosity of *Apc* in the *Apc* mutant mice, however, the organoids exposed to ETBF showed near identical mutational profiles to unexposed controls, suggesting that ETBF does not cause a unique mutational signature unlike the SBS88 and ID18 signatures associated with *pks*^*+*^ *E. coli* [60]. This suggests that the carcinogenic mechanism of ETBF may not involve characteristic genomic aberrations and suggests alternative mechanisms including DNA methylation. Maiuri et al. examined genome-wide DNA methylation in mice exposed to ETBF. The authors reported that normal epithelium exposed to ETBF undergoes inflammation-driven tumorigenesis and caused a unique DNA methylation signature [61]. Interestingly, these methylation aberrations were abrogated in mice with dysfunctional *Msh2*, however, yet still promoting tumorigenesis. This suggests a multi-faceted role of ETBF and warrants further investigation of ETBF as the tumorigenic instigator and the risk mediator via modifying the epigenome.

### F. nucleatum

*F. nucleatum* was associated with tumour characteristics related to MMRd and CRCs of the serrated pathway (e.g., right location, *BRAF* p.V600E, CIMP-high), consistent with previous reports [62, 63]. In addition, our study identified that *F. nucleatum* was associated with MMRd related to Lynch syndrome as well as sporadic MMRd CRCs related to *MLH1* promoter methylation and double MMR somatic mutations. These findings suggest that *F. nucleatum* colonisation in the tumour is not related to MMRd etiology. This supports *F. nucleatum* causing opportunistic infections [64], exploiting the specific tumour microenvironment of MMRd CRCs.

Though *F. nucleatum* is found to be highly abundant in CRC tumours [64–66], the oncogenic mechanism of *F. nucleatum* remains to be elucidated, especially lacking are studies investigating their effect on the host genetic and epigenetics. Our study identified an association between TILs and *F. nucleatum* and this suggests the favourable tumour microenvironment of *F. nucleatum*. Given this association was no longer significant in the stratified analysis by MMR status, it may suggest that this interrelationship is dependent from the overrepresentation of TILs in MMRd CRCs, rather than suggesting a causative role of *F. nucleatum*.

### Strengths and weaknesses of this study

Our study has several strengths including a large sample size from three CRC cohorts that have extensive molecular characterisation. Our study was the first to screen for intratumoral presence of these three CRC-associated bacteria but also to investigate the association between *pks*^*+*^ *E. coli* and the *APC*: c.835-8 A > G somatic mutation that is mechanistically linked to colibactin-related DNA damage. Our study also provided a stratified analysis of clinically relevant MMRd subgroups of both hereditary and sporadic etiologies, providing further insight into the nature of the association between MMRd and these bacteria. Further, by including a large number of EOCRCs, our study has provided separate analysis on EOCRCs, which is an emerging health problem, globally [67]. A further strength of our study was the use of assays that targeted both the *pks* island and *E. coli*, ensuring differentiation *pks*^*+*^ *E. coli*^*+*^ from other *pks* harbouring bacteria. This differentiation highlighted the specific association between *pks*^*+*^ *E. coli*^*+*^ and the *APC*: c.835-8 A > G somatic mutation.

The limitations of this study include the cross-sectional study design where in our participants, the prior infection of these bacteria could not be examined. Ex vivo or in vitro studies such as organoids co-culture experiments [19, 60] may further elucidate the direct effect these bacteria may have on colonic mucosa. Additionally, bacterial screening on premalignant polyps may help strengthen the early role of these bacteria in driving colorectal neoplasm.

In this study, we utilised FFPE specimens for detecting intratumoral bacteria. Although this biospecimen type is commonly used in such studies [12, 56], a recent meta-analysis indicated that the biospecimen type could influence the detection efficacy and affect the results [68]. Further studies performed on different biospecimen types (e.g., fresh frozen) may be needed to validate the findings from our study. Another limitation includes the lack of TNM stage information.

## Conclusion

This study provides novel findings on specific molecular features and pathways of tumorigenesis associated with each genotoxic gut bacterium. The strength of the association between the presence of intratumoral *pks*^*+*^ *E. coli*^*+*^ and *APC*: c.835-8 A > G somatic mutation is shown for the first time. This has important clinical implication as the *APC*: c.835-8 A > G somatic mutation may represent a biomarker for colibactin-induced DNA damage in CRC tumours caused by *pks*^*+*^ *E. coli*^*+*^. This finding provides new opportunities for future studies on prevention and treatments of bacterial-driven CRCs.

### Supplementary information

Supplementary Figure Legends

Supplementary Table 1

Supplementary Table 2

Supplementary Table 3

Supplementary Table 4

Supplementary Methods

Supplementary Figure 1

Supplementary Figure 2

## Data Availability

The data and materials presented in this publication were collected according to the guidelines of the Australian Colon Cancer Family Registry, Melbourne Collaborative Cohort Study and the ANGELS studies. The data generated in this study can be accessed via request to the Colon Cancer Family Registry (https://www.coloncfr.org/collaboration) or via contacting the corresponding author (DDB) and study investigators for the MCCS and ANGELS studies.

## References

[1] Sung H, Ferlay J, Siegel RL, Laversanne M, Soerjomataram I, Jemal A (2021). Global cancer statistics 2020: GLOBOCAN estimates of incidence and mortality worldwide for 36 cancers in 185 countries. CA Cancer J Clin.

[2] Australian Institute of Health and Welfare 2017. Cancer in Australia 2017. Cancer series no.101. Cat. no. CAN 100. Canberra: AIHW.

[3] Jass JR (2007). Classification of colorectal cancer based on correlation of clinical, morphological and molecular features. Histopathology.

[4] He X, Wu K, Ogino S, Giovannucci EL, Chan AT, Song M (2018). Association between risk factors for colorectal cancer and risk of serrated polyps and conventional adenomas. Gastroenterology.

[5] Louis P, Hold GL, Flint HJ (2014). The gut microbiota, bacterial metabolites and colorectal cancer. Nat Rev Microbiol.

[6] Grady WM (2003). Genetic testing for high-risk colon cancer patients. Gastroenterology.

[7] Yu J, Feng Q, Wong SH, Zhang D, Liang QY, Qin Y (2017). Metagenomic analysis of faecal microbiome as a tool towards targeted non-invasive biomarkers for colorectal cancer. Gut.

[8] Nakatsu G, Li X, Zhou H, Sheng J, Wong SH, Wu WK (2015). Gut mucosal microbiome across stages of colorectal carcinogenesis. Nat Commun.

[9] Sobhani I, Tap J, Roudot-Thoraval F, Roperch JP, Letulle S, Langella P (2011). Microbial dysbiosis in colorectal cancer (CRC) patients. PLoS One.

[10] Iyadorai T, Mariappan V, Vellasamy KM, Wanyiri JW, Roslani AC, Lee GK (2020). Prevalence and association of pks+ Escherichia coli with colorectal cancer in patients at the University Malaya Medical Centre, Malaysia. PLoS One (Artic).

[11] Zhou Y, He H, Xu H, Li Y, Li Z, Du Y (2016). Association of oncogenic bacteria with colorectal cancer in South China. Oncotarget.

[12] Mima K, Sukawa Y, Nishihara R, Qian ZR, Yamauchi M, Inamura K (2015). Fusobacterium nucleatum and T cells in colorectal carcinoma. JAMA Oncol.

[13] Clermont O, Bonacorsi S, Bingen E (2000). Rapid and simple determination of the Escherichia coli phylogenetic group. Appl Environ Microbiol.

[14] Nougayrede JP, Homburg S, Taieb F, Boury M, Brzuszkiewicz E, Gottschalk G (2006). Escherichia coli induces DNA double-strand breaks in eukaryotic cells. Science.

[15] Arthur JC, Perez-Chanona E, Muhlbauer M, Tomkovich S, Uronis JM, Fan TJ (2012). Intestinal inflammation targets cancer-inducing activity of the microbiota. Science.

[16] Xue M, Kim CS, Healy AR, Wernke KM, Wang Z, Frischling MC et al. Structure elucidation of colibactin and its DNA cross-links. Science. 2019;365. 10.1126/science.aax2685. 10.1126/science.aax2685 PMC6820679 31395743

[17] Bossuet-Greif N, Vignard J, Taieb F, Mirey G, Dubois D, Petit C et al. The colibactin genotoxin generates DNA interstrand cross-links in infected cells. mBio. 2018;9. 10.1128/mBio.02393-17. 10.1128/mBio.02393-17 PMC5874909 29559578

[18] Iftekhar A, Berger H, Bouznad N, Heuberger J, Boccellato F, Dobrindt U (2021). Genomic aberrations after short-term exposure to colibactin-producing E. coli transform primary colon epithelial cells. Nat Commun.

[19] Pleguezuelos-Manzano C, Puschhof J, Rosendahl Huber A, van Hoeck A, Wood HM, Nomburg J (2020). Mutational signature in colorectal cancer caused by genotoxic pks(+) E. coli. Nature.

[20] Terlouw D, Suerink M, Boot A, van Wezel T, Nielsen M, Morreau H (2020). Recurrent APC splice variant c.835-8A>G in patients with unexplained colorectal polyposis fulfilling the colibactin mutational signature. Gastroenterology.

[21] Sears CL, Islam S, Saha A, Arjumand M, Alam NH, Faruque AS (2008). Association of enterotoxigenic Bacteroides fragilis infection with inflammatory diarrhea. Clin Infect Dis Off Publ Infect Dis Soc Am.

[22] Wu S, Rhee KJ, Albesiano E, Rabizadeh S, Wu X, Yen HR (2009). A human colonic commensal promotes colon tumorigenesis via activation of T helper type 17 T cell responses. Nat Med.

[23] Rubinstein MR, Wang X, Liu W, Hao Y, Cai G, Han YW (2013). Fusobacterium nucleatum promotes colorectal carcinogenesis by modulating E-cadherin/β-catenin signaling via its FadA adhesin. Cell Host Microbe.

[24] Gur C, Ibrahim Y, Isaacson B, Yamin R, Abed J, Gamliel M (2015). Binding of the Fap2 protein of Fusobacterium nucleatum to human inhibitory receptor TIGIT protects tumors from immune cell attack. Immunity.

[25] Kostic AD, Chun E, Robertson L, Glickman JN, Gallini CA, Michaud M (2013). Fusobacterium nucleatum potentiates intestinal tumorigenesis and modulates the tumor-immune microenvironment. Cell Host Microbe.

[26] Newcomb PA, Baron J, Cotterchio M, Gallinger S, Grove J, Haile R (2007). Colon Cancer Family Registry: an international resource for studies of the genetic epidemiology of colon cancer. Cancer Epidemiol Biomark Prev.

[27] Jenkins MA, Win AK, Templeton AS, Angelakos MS, Buchanan DD, Cotterchio M et al. Cohort profile: the colon cancer family registry cohort (CCFRC). Int J Epidemiol. 2018. 10.1093/ije/dyy006. 10.1093/ije/dyy006 PMC5913593 29490034

[28] Buchanan DD, Clendenning M, Rosty C, Eriksen SV, Walsh MD, Walters RJ (2017). Tumor testing to identify Lynch syndrome in two Australian colorectal cancer cohorts. J Gastroenterol Hepatol.

[29] Giles GG, English DR (2002). The Melbourne collaborative cohort study. IARC Sci Publ.

[30] Milne RL, Fletcher AS, MacInnis RJ, Hodge AM, Hopkins AH, Bassett JK (2017). Cohort profile: the Melbourne collaborative cohort study (Health 2020). Int J Epidemiol.

[31] Buchanan DD, Rosty C, Clendenning M, Spurdle AB, Win AK (2014). Clinical problems of colorectal cancer and endometrial cancer cases with unknown cause of tumor mismatch repair deficiency (suspected Lynch syndrome). Appl Clin Genet.

[32] Georgeson P, Pope BJ, Rosty C, Clendenning M, Mahmood K, Joo JE (2021). Evaluating the utility of tumour mutational signatures for identifying hereditary colorectal cancer and polyposis syndrome carriers. Gut (Artic).

[33] Walker R, Georgeson P, Mahmood K, Joo JE, Makalic E, Clendenning M (2023). Evaluating multiple next-generation sequencing–derived tumor features to accurately predict DNA mismatch repair status. J Mol Diagn.

[34] Rosty C, Young JP, Walsh MD, Clendenning M, Walters RJ, Pearson S (2013). Colorectal carcinomas with KRAS mutation are associated with distinctive morphological and molecular features. Mod Pathol.

[35] Jenkins MA, Hayashi S, O’Shea AM, Burgart LJ, Smyrk TC, Shimizu D (2007). Pathology features in Bethesda guidelines predict colorectal cancer microsatellite instability: a population-based study. Gastroenterology.

[36] Lindor NM, Burgart LJ, Leontovich O, Goldberg RM, Cunningham JM, Sargent DJ (2002). Immunohistochemistry versus microsatellite instability testing in phenotyping colorectal tumors. J Clin Oncol.

[37] Cicek MS, Lindor NM, Gallinger S, Bapat B, Hopper JL, Jenkins MA (2011). Quality assessment and correlation of microsatellite instability and immunohistochemical markers among population- and clinic-based colorectal tumors results from the Colon Cancer Family Registry. J Mol Diagn.

[38] Walsh MD, Buchanan DD, Cummings MC, Pearson SA, Arnold ST, Clendenning M (2010). Lynch syndrome-associated breast cancers: clinicopathologic characteristics of a case series from the colon cancer family registry. Clin Cancer Res.

[39] Walker R, Mahmood K, Joo JE, Clendenning M, Georgeson P, Como J (2023). A tumor focused approach to resolving the etiology of DNA mismatch repair deficient tumors classified as suspected Lynch syndrome. J Transl Med.

[40] Poynter JN, Siegmund KD, Weisenberger DJ, Long TI, Thibodeau SN, Lindor N (2008). Molecular characterization of MSI-H colorectal cancer by MLHI promoter methylation, immunohistochemistry, and mismatch repair germline mutation screening. Cancer Epidemiol Biomark Prev.

[41] Clendenning M, Walsh MD, Gelpi JB, Thibodeau SN, Lindor N, Potter JD (2013). Detection of large scale 3′ deletions in the PMS2 gene amongst Colon-CFR participants: have we been missing anything?. Fam Cancer.

[42] Buchanan DD, Tan YY, Walsh MD, Clendenning M, Metcalf AM, Ferguson K (2014). Tumor mismatch repair immunohistochemistry and DNA MLH1 methylation testing of patients with endometrial cancer diagnosed at age younger than 60 years optimizes triage for population-level germline mismatch repair gene mutation testing. J Clin Oncol.

[43] Pope BJ, Clendenning M, Rosty C, Mahmood K, Georgeson P, Joo JE (2021). Germline and tumor sequencing as a diagnostic tool to resolve suspected Lynch syndrome. J Mol Diagn.

[44] Rosty C, Buchanan DD, Walsh MD, Pearson SA, Pavluk E, Walters RJ (2012). Phenotype and polyp landscape in serrated polyposis syndrome: a series of 100 patients from genetics clinics. Am J Surg Pathol.

[45] Buchanan DD, Sweet K, Drini M, Jenkins MA, Win AK, English DR (2010). Risk factors for colorectal cancer in patients with multiple serrated polyps: a cross-sectional case series from genetics clinics. PloS One.

[46] Weisenberger DJ, Siegmund KD, Campan M, Young J, Long TI, Faasse MA (2006). CpG island methylator phenotype underlies sporadic microsatellite instability and is tightly associated with BRAF mutation in colorectal cancer. Nat Genet.

[47] Sherry NL, Lee RS, Gorrie CL, Kwong JC, Stuart RL, Korman TM (2021). Pilot study of a combined genomic and epidemiologic surveillance program for hospital-acquired multidrug-resistant pathogens across multiple hospital networks in Australia. Infect Control Hosp Epidemiol.

[48] Sherry NL, Lee RS, Gorrie CL, Kwong JC, Stuart RL, Korman T et al. Genomic interrogation of the burden and transmission of multidrug-resistant pathogens within and across hospital networks. 2019:764787; 10.1101/764787%JbioRxiv.

[49] Buc E, Dubois D, Sauvanet P, Raisch J, Delmas J, Darfeuille-Michaud A (2013). High prevalence of mucosa-associated E. coli producing cyclomodulin and genotoxin in colon cancer. PloS One.

[50] Bonnet M, Buc E, Sauvanet P, Darcha C, Dubois D, Pereira B (2014). Colonization of the human gut by E. coli and colorectal cancer risk. Clin Cancer Res.

[51] Tsunematsu Y, Hosomi K, Kunisawa J, Sato M, Shibuya N, Saito E (2021). Mother-to-infant transmission of the carcinogenic colibactin-producing bacteria. BMC Microbiol.

[52] Lee-Six H, Olafsson S, Ellis P, Osborne RJ, Sanders MA, Moore L (2019). The landscape of somatic mutation in normal colorectal epithelial cells. Nature.

[53] Arrieta MC, Stiemsma LT, Amenyogbe N, Brown EM, Finlay B (2014). The intestinal microbiome in early life: health and disease. Front Immunol.

[54] Boot A, Ng AWT, Chong FT, Ho SC, Yu W, Tan DSW (2020). Characterization of colibactin-associated mutational signature in an Asian oral squamous cell carcinoma and in other mucosal tumor types. Genome Res.

[55] Yaeger R, Chatila WK, Lipsyc MD, Hechtman JF, Cercek A, Sanchez-Vega F (2018). Clinical sequencing defines the genomic landscape of metastatic colorectal cancer. Cancer Cell.

[56] Arima K, Zhong R, Ugai T, Zhao M, Haruki K, Akimoto N (2022). Western-style diet, pks island-carrying escherichia coli, and colorectal cancer: analyses from two large prospective cohort studies. Gastroenterology.

[57] Rabizadeh S, Rhee KJ, Wu S, Huso D, Gan CM, Golub JE (2007). Enterotoxigenic bacteroides fragilis: a potential instigator of colitis. Inflamm Bowel Dis.

[58] Dejea CM, Fathi P, Craig JM, Boleij A, Taddese R, Geis AL (2018). Patients with familial adenomatous polyposis harbor colonic biofilms containing tumorigenic bacteria. Science.

[59] Chung L, Orberg ET, Geis AL, Chan JL, Fu K, DeStefano Shields CE (2018). Bacteroides fragilis toxin coordinates a pro-carcinogenic inflammatory cascade via targeting of colonic epithelial cells. Cell Host Microbe.

[60] Allen J, Rosendahl Huber A, Pleguezuelos-Manzano C, Puschhof J, Wu S, Wu X (2022). Colon tumors in enterotoxigenic bacteroides fragilis (ETBF)-colonized mice do not display a unique mutational signature but instead possess host-dependent alterations in the APC gene. Microbiol Spectr.

[61] Maiuri AR, Peng M, Podicheti R, Sriramkumar S, Kamplain CM, Rusch DB (2017). Mismatch repair proteins initiate epigenetic alterations during inflammation-driven tumorigenesis. Cancer Res.

[62] Mima K, Nishihara R, Qian ZR, Cao Y, Sukawa Y, Nowak JA (2016). Fusobacterium nucleatum in colorectal carcinoma tissue and patient prognosis. Gut.

[63] Tahara T, Yamamoto E, Suzuki H, Maruyama R, Chung W, Garriga J (2014). Fusobacterium in colonic flora and molecular features of colorectal carcinoma. Cancer Res.

[64] Brennan CA, Garrett WS (2019). Fusobacterium nucleatum—symbiont, opportunist and oncobacterium. Nat Rev Microbiol.

[65] Song M, Chan AT, Sun J (2020). Influence of the gut microbiome, diet, and environment on risk of colorectal cancer. Gastroenterology.

[66] Castellarin M, Warren RL, Freeman JD, Dreolini L, Krzywinski M, Strauss J (2012). Fusobacterium nucleatum infection is prevalent in human colorectal carcinoma. Genome Res.

[67] Ugai T, Sasamoto N, Lee HY, Ando M, Song M, Tamimi RM (2022). Is early-onset cancer an emerging global epidemic? Current evidence and future implications. Nat Rev Clin Oncol.

[68] Kim Y, Cho NY, Kang GH (2022). Prognostic and clinicopathological significance of Fusobacterium nucleatum in colorectal cancer: a systemic review and meta-analysis. J Pathol Transl Med.

