# Supplementary material for: Intratumoral presence of the genotoxic gut bacteria pks+ E. coli, Enterotoxigenic Bacteroides fragilis, and Fusobacterium nucleatum and their association with clinicopathological and molecular features of colorectal cancer
Source: Br J Cancer. 2024 Jan 10;130(5):728–40. doi: 10.1038/s41416-023-02554-x (PMC10912205; doi:10.1038/s41416-023-02554-x)
Supplement: Supplementary file 1 — Supplementary Figure Legends [file 41416_2023_2554_MOESM1_ESM.docx]

**Title: Intratumoral presence of the genotoxic gut bacteria *pks+ E. coli*, Enterotoxigenic *Bacteroides fragilis,* and *Fusobacterium nucleatum* and their association with clinicopathological and molecular features of colorectal cancer**

**Supplementary Figure Legends**

**Supplementary Figure 1** Colorectal cancers (CRCs) from Melbourne Collaborative Cohort Study (MCCS), Australasian Colon Cancer Family Registry (ACCFR) and Applying Novel Genomic approaches to Early-onset and suspected Lynch Syndrome colorectal and endometrial cancers (ANGELS) study that were included in the testing of intratumoral *pks^+^ E. coli^+^*, *pks^+^ E. coli^-^*, ETBF and *F. nucleatum*.

**Supplementary Figure 2** Venn diagram illustrating the co-occurrence of *pks^+^ E. coli^+^,* ETBF and *F. nucleatum* in 1697 CRCs included in this study.
