# Supplementary material for: Intratumoral presence of the genotoxic gut bacteria pks+ E. coli, Enterotoxigenic Bacteroides fragilis, and Fusobacterium nucleatum and their association with clinicopathological and molecular features of colorectal cancer
Source: Br J Cancer. 2024 Jan 10;130(5):728–40. doi: 10.1038/s41416-023-02554-x (PMC10912205; doi:10.1038/s41416-023-02554-x)
Supplement: Supplementary file 6 — Supplementary Methods [file 41416_2023_2554_MOESM6_ESM.docx]

**Supplementary Methods**

**Title: Intratumoral presence of the genotoxic gut bacteria *pks+ E. coli*, Enterotoxigenic *Bacteroide fragilis,* and *Fusobacterium nucleatum* and their association with clinicopathological and molecular features of colorectal cancer**

**Quantitative polymerase chain (qPCR) assays for detecting *pks^+^ E. coli*, ETBF and *F. nucleatum***

The intratumoural presence of *pks^+^ E. coli*, ETBF, and *F. nucleatum* was assessed by performing quantitative polymerase chain reaction (qPCR) targeting four bacterial genes. For the detection of the *pks+ E. coli*, SYBR Green assays targeting the *ClbB* (*pks* island) and the *UidA* (*E. coli*) genes were used previously described ^1^, with *ClbB* primers modified as follows: forward primer, 5’-CGACCGTCGAATAGCGTTGGAAT-3’; and reverse primer, 5’-GCTCTATGCTCATCAACCCATGT-3’. The human housekeeping gene, β-actin, was used as an internal control as previously described ^2^. Each PCR reaction was performed in technical duplicates and contained 20ng of genomic DNA in a 10µL reaction comprising 1 x final concentration POWER SYBR Green PCR Master Mix (Thermo Fisher Scientific, California, USA). The qPCR was performed on Thermo QuantStudio 7 (Thermo Fisher Scientific, California, USA). Positive and negative controls namely, *E. coli* strains known to contain the *pks* island and without the *pks* island were included. Based on the amplification of *ClbB* (the pks island) and *UidA* (*E. coli*), each tumour DNA sample was catergorised into four groups:

1. *Pks+ E. coli+* showing the amplification of both *ClbB* and *UidA* genes, indicating the presence of only *E. coli* with *pks* island.
2. *Pks+ E. coli-* showing the amplification of the *ClbB* gene but not *UidA*, indicating the presence of bacteria with *pks* island that are not *E. coli*.
3. *Pks+ (all bacteria)* includes all tuomur samples showing the amplification of the *ClbB* gene, indicating any bacteria with *pks* island.
4. *Pks- E. coli+* showing the ampliation of *UidA* only, indicating the presence of *E. coli* without *pks* island.

For the detection of ETBF and *F. nucleatum*, Taqman assays targeting the *Bft* ^3,4^ and *nusG* ^5^ genes were used, respectively and the assays were run along the human housekeeping gene, *SLCO2A1*, used as the internal control as previously described ^5^. Positive and negative controls were obtained from Leibniz-Institute DSMZ (Braunschweig, Germany). Each TaqMan reaction was performed in technical duplicates. In each qPCR, 20ng of genomic DNA was used in a 10µL reaction comprising 1 x final concentration TaqMan Universal Master Mix (Thermo Fisher Scientific, California, USA) in a 384-well optical PCR plate (Thermo Fisher Scientific). qPCR was performed on Thermo QuantStudio 7 (Thermo Fisher Scientific).

A melt curve was used to determine the specificity of the amplicon. The presence of *pks* island and *E. coli* were detected using the standard cycle threshold (Ct) and melt curve analysis on QuantStudio 7 (QS7) software (Thermo Fisher Scientific, California, USA). An independent TaqMan assay targeting the *ClbB* gene was used as previously described ^6^ in a subset of tumors to confirm results from the SYBR Green assay. Each PCR reaction was performed in technical duplicates and contained 20ng of genomic DNA in a 10µL reaction comprising 1 x final concentration TaqMan Environmental Master Mix (Thermo Fisher Scientific, California, USA) set up in a 384-well optical PCR plate (Thermo Fisher Scientific) in the same conditions used as per the SYBR *pks E. coli* assay. The presence of *pks* island was detected using the standard Ct calculation, performed using the QS7 software (Thermo Fisher Scientific). The average ΔCt (the average Ct value of bacteria – the average Ct value of human housekeeping gene) was 15 for *pks+ E. coli and* ETBF. The average ΔCt was 24 for *F. nucleatum* positive CRCs.

**References**

1. Shimpoh T, Hirata Y, Ihara S, Suzuki N, Kinoshita H, Hayakawa Y *et al.* Prevalence of pks-positive Escherichia coli in Japanese patients with or without colorectal cancer. *Gut Pathog* 2017; **9**: 35; e-pub ahead of print 2017/06/16; doi 10.1186/s13099-017-0185-x.

2. Bundgaard-Nielsen C, Baandrup UT, Nielsen LP, Sørensen S. The presence of bacteria varies between colorectal adenocarcinomas, precursor lesions and non-malignant tissue. *BMC cancer* 2019; **19**(1): 399; e-pub ahead of print 2019/05/01; doi 10.1186/s12885-019-5571-y.

3. Chen LA, Van Meerbeke S, Albesiano E, Goodwin A, Wu S, Yu H *et al.* Fecal detection of enterotoxigenic Bacteroides fragilis. *European journal of clinical microbiology & infectious diseases : official publication of the European Society of Clinical Microbiology* 2015; **34**(9): 1871-1877; doi 10.1007/s10096-015-2425-7.

4. Purcell RV, Pearson J, Frizelle FA, Keenan JI. Comparison of standard, quantitative and digital PCR in the detection of enterotoxigenic Bacteroides fragilis. *Scientific reports* 2016; **6**: 34554; e-pub ahead of print 2016/10/01; doi 10.1038/srep34554.

5. Mima K, Sukawa Y, Nishihara R, Qian ZR, Yamauchi M, Inamura K *et al.* Fusobacterium nucleatum and T Cells in Colorectal Carcinoma. *JAMA oncology* 2015; **1**(5): 653-661; doi 10.1001/jamaoncol.2015.1377.

6. Arima K, Zhong R, Ugai T, Zhao M, Haruki K, Akimoto N *et al.* Western-Style Diet, Polyketide Synthase (pks) Island-Carrying Escherichia coli, and Colorectal Cancer: Analyses From Two Large Prospective Cohort Studies. *Gastroenterology* 2022; e-pub ahead of print 2022/06/28; doi 10.1053/j.gastro.2022.06.054.
