# Supplementary figures and images for: Intratumoral presence of the genotoxic gut bacteria pks+ E. coli, Enterotoxigenic Bacteroides fragilis, and Fusobacterium nucleatum and their association with clinicopathological and molecular features of colorectal cancer
Source: Br J Cancer. 2024 Jan 10;130(5):728–40. doi: 10.1038/s41416-023-02554-x (PMC10912205; doi:10.1038/s41416-023-02554-x)

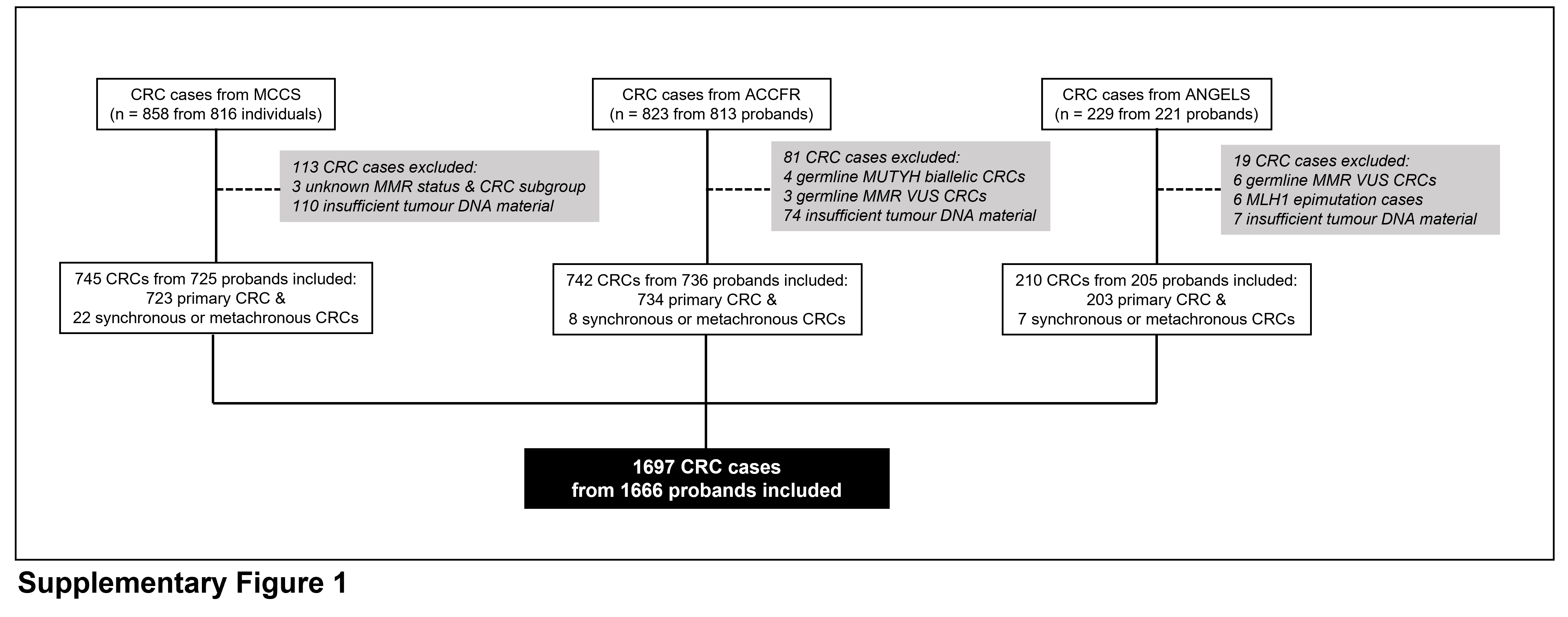

Supplement: Supplementary file 7 — Supplementary Figure 1 [file 41416_2023_2554_MOESM7_ESM.png]

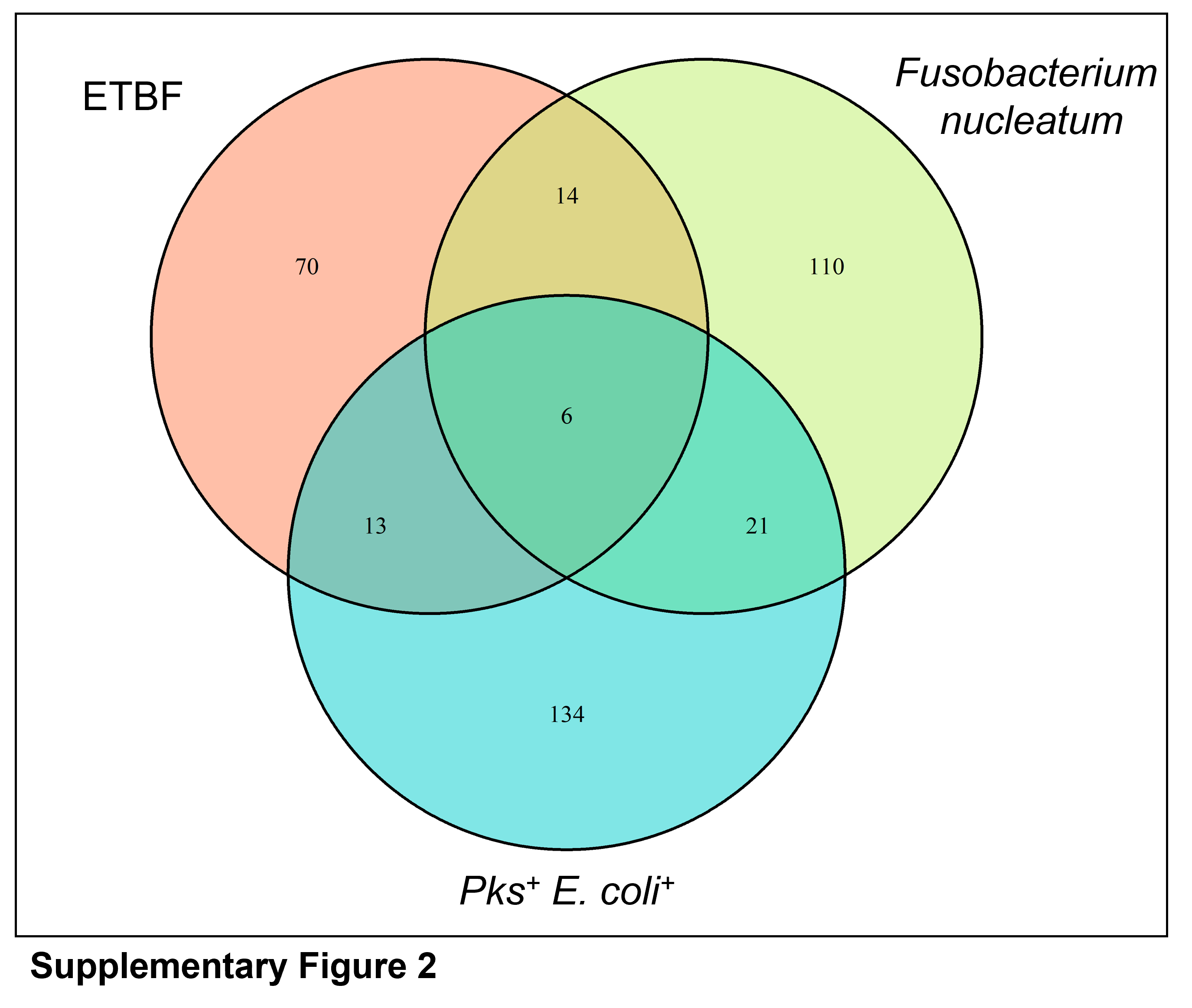

Supplement: Supplementary file 8 — Supplementary Figure 2 [file 41416_2023_2554_MOESM8_ESM.png]
